# V2N-Based Comprehensive Safety Framework by Prediction of VRU Movement on Community Roads with Management of Route Branching at Intersections

**DOI:** 10.3390/s26041229

**Published:** 2026-02-13

**Authors:** Kota Watanabe, Takuma Ito

**Affiliations:** Graduate School of Engineering, The University of Tokyo, 7-3-1 Hongo, Bunkyo, Tokyo 113-8656, Japan

**Keywords:** movement estimation, Vulnerable Road User, Extended Kalman Filter, cooperative safety, V2N

## Abstract

Traffic accidents involving Vulnerable Road Users (VRUs) frequently occur at unsignalized intersections on Japanese community roads. To prevent such accidents, collision avoidance systems need to predict VRUs’ movements throughout the entire road network while explicitly handling uncertainty degraded by sparse observations and frequent route branching at intersections. Based on this motivation, this study proposes a Vehicle-to-Network (V2N)-based comprehensive safety framework for estimation of VRU movement and prediction of future intersection entry for community roads. The framework integrates estimation results provided from Roadside Edges and Vehicle Edges at a Central Server. In addition, road geometry from map information is incorporated as pseudo-observations into the estimation, and multiple route hypotheses are explicitly managed to represent route branching at intersections. For intersection-entry prediction, entry certainty is calculated by integrating a predicted distribution. For evaluation of the proposed framework, we conduct Monte Carlo simulations on simplified grid road networks. The results show that the proposed framework maintains conservative estimation under sparse observations and improves prediction when additional observation information from surrounding vehicles becomes available. Furthermore, a simulation-based case study using an actual community road-network geometry shows the feasibility of the proposed framework for cooperative collision avoidance on actual community roads.

## 1. Introduction

**Figure 1:**
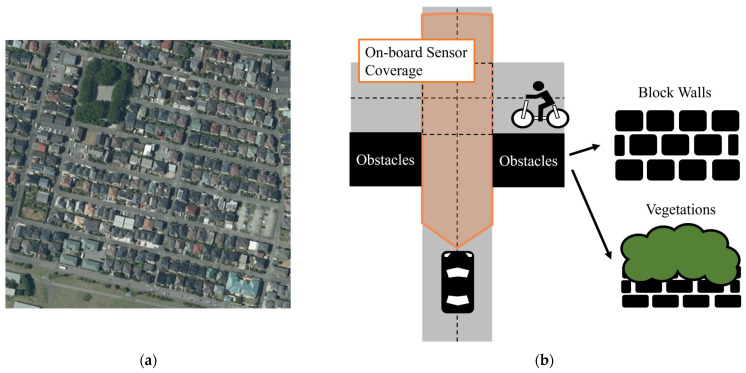
(**a**) Aerial photo of a general community road (created by editing the digital map and aerial photograph [2]). (**b**) Typical environment at unsignalized intersections on community roads.

In Japan, traffic accidents on community roads have not been sufficiently resolved [1]. These roads frequently have narrow road width and mixed traffic with Vulnerable Road Users (VRUs). Figure 1a,b show an aerial photo of a general community road [2] and a typical environment at unsignalized intersections, respectively. As shown in Figure 1a, these roads contain many unsignalized intersections. In addition, obstacles such as block walls and vegetation limit the visibility there, as shown in Figure 1b. Due to such unfavorable characteristics, automobile drivers frequently fail to detect VRUs at unsignalized intersections, and collisions occur when VRUs suddenly enter the intersection. To address this issue, widespread collision avoidance systems that rely only on a vehicle’s on-board sensors are insufficient because they cannot recognize environments outside the sensor range. Therefore, to prevent collisions involving VRUs at unsignalized intersections on community roads, more comprehensive safety functions based on cooperative and connected approaches are necessary. In this study, we focused on Vehicle-to-Network (V2N)-based collision avoidance systems, which can recognize environments over wide areas by integrating provided information.

**Figure 2:**
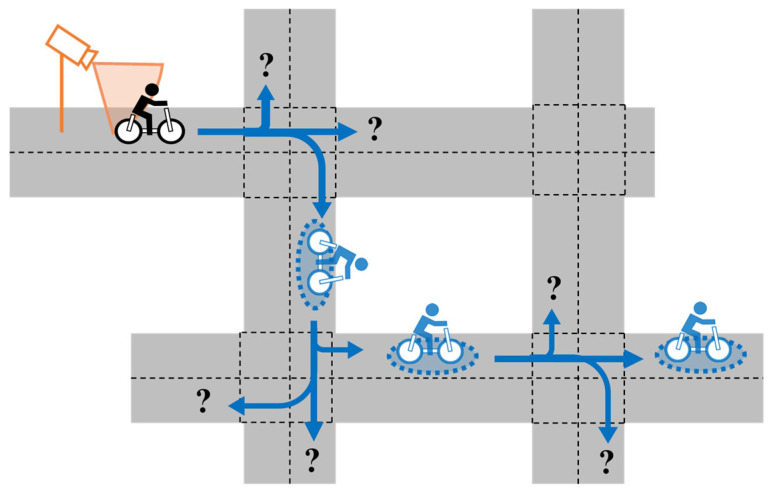
Typical situation where a VRU’s future route becomes ambiguous due to multiple intersections on community roads.

Although collision avoidance systems need to predict VRUs’ movements for collision prevention at intersections, they are limited by two difficulties on community roads. First, available observation information from sensors is sparse. For predicting VRUs’ movements based on sensor observations, it is ideal to place roadside sensors at all intersections. However, this is not feasible due to installation and maintenance costs, and only sparse observation information is available across the road network. Second, route branching at intersections occurs frequently. Figure 2 shows a typical situation where a VRU’s future route becomes ambiguous due to multiple intersections. Because residential areas are often developed in a grid-like layout, community roads have many intersections. As a result, the future route of the VRU becomes ambiguous at intersections. These two difficulties limit opportunities to observe VRUs and limit the effect of each observation only until the VRU reaches the next intersection. In other words, the systems need to explicitly address the degradation of the prediction uncertainty caused by sparse observations and frequent route branching. However, most existing studies on V2N-based collision avoidance systems focus on functions at highways or single intersections, not entire road networks. Therefore, a framework that can handle the degradation of prediction uncertainty over an entire community road network is required.

Based on the above considerations, we aim to develop V2N-based technology for VRU movement prediction on community roads under sparse observations and frequent route branching at intersections. Here, the important point is not to consistently provide accurate warnings to each individual vehicle, but to avoid missed warnings against hazardous situations involving VRUs at the road-network level. On community roads, observation information is spatially and temporally sparse, which results in unavoidable limitations on the available information. Under such conditions, aggregating sparse information from vehicles and roadside units can support continuous estimation of VRU movements over the road network, even though each estimation at the level of individual vehicles is imperfect. From this perspective, the occurrence of false warnings that are unnecessary for individual vehicles is less critical than the occurrence of missed warnings of a dangerous situation for VRUs at the road-network level. Therefore, our primary objective is to reduce missed warnings at the road-network level, and subsequently to improve warning accuracy at the level of individual vehicles while preserving network-level safety.

**Figure 3:**
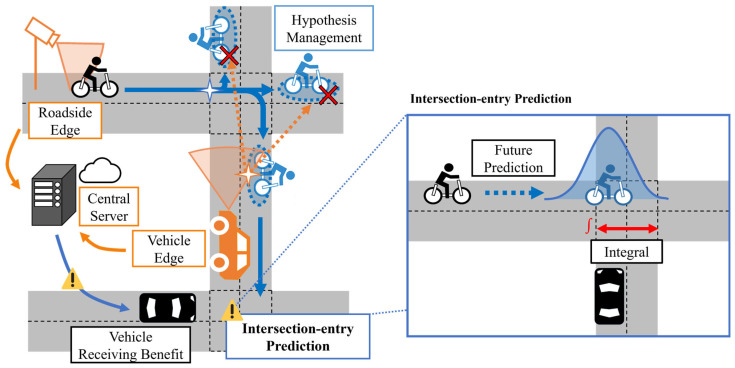
Conceptual diagram of assumed situation.

With this objective, this study proposes a V2N-based framework for predicting VRU movement and future entry into an intersection. Figure 3 illustrates the conceptual diagram of the assumed situation. The framework consists of Roadside Edges, Vehicle Edges, and a Central Server. The Server integrates estimations from Edges and explicitly manages route branching at intersections as multiple route hypotheses to predict future movement. Based on the predicted intersection-entry certainty, the Central Server predicts the future entry of VRUs into intersections, which will be used for collision avoidance. In addition to the above system design, we evaluate the proposed framework through simulation experiments on community road networks. Through these simulations, we analyze the feasibility and practical usefulness of the proposed framework for community road networks.

The main contributions of this study are as follows:

- Proposal of cooperative framework that predicts VRU movement and intersection entry with sparse observations and frequent route branching.
- Analysis of the framework’s usefulness against the availability of observation information through Monte Carlo simulations.

The remainder of this paper is organized as follows: Section 2 organizes related work of V2N-based systems for VRU safety. Section 3 explains the details of the proposed framework. Section 4 describes the design of simulation experiments and Section 5 analyzes the results. Section 6 organizes the discussion and limitations of this study. Finally, Section 7 summarizes the conclusions and explains the future work.

## 2. Related Work

This section reviews existing studies related to V2N-based systems and stochastic prediction methods for VRU safety. First, Section 2.1 summarizes V2N-based cooperative safety systems that integrate information from vehicles, roadside sensors, and smartphones. We describe their limitations in terms of spatial coverage over community road networks. Next, Section 2.2 reviews stochastic prediction methods for collision prevention. We organize stochastic motion estimation, behavior prediction, and collision risk evaluation. Then, Section 2.3 organizes methods of cooperative prediction using multiple information sources. Finally, we review approaches that incorporate road geometry and route branching into position estimation in Section 2.4. We emphasize that the management of route branching has not been sufficiently investigated in V2N-based frameworks that explicitly integrate information from multiple distributed sources.

### 2.1. Cooperative Safety System for VRU Safety

With the advancement of wireless communication technologies, research on cooperative systems for VRU safety has been widely addressed [3]. Among cooperative safety systems for VRUs, one approach is to use VRUs’ smartphones as both communication devices and sources of location information obtained from Global Navigation Satellite System (GNSS) data. Several studies have demonstrated the feasibility of Vehicle-to-Pedestrian systems utilizing VRUs’ smartphones. Dhondge et al. [4] proposed a system that obtains GNSS and Inertial Measurement Unit (IMU) data from VRUs and vehicle drivers’ smartphones to send collision warnings. Hussein et al. [5] developed a mobile application that predicts collisions by combining vehicles’ information with VRUs’ GNSS data. Additionally, Liu et al. [6] developed a system that predicts collisions using roadside radar data and smartphone GNSS data from VRUs and vehicles, and sends warning messages to smartphones. As shown by the above studies, smartphones provide a useful role in cooperative systems for VRU safety. However, they share the assumption that VRUs continuously carry smartphones and provide information to the system. This assumption limits the applicability of such approaches for VRUs who do not carry smartphones or do not provide their information to the system in real-world environments. From this viewpoint, safety systems that do not rely only on smartphone data from VRUs are also required.

The above limitation of reliance on VRUs’ smartphones motivates the development of V2N-based systems that comprehensively integrate information from vehicles and roadside sensors in addition to smartphones. These V2N-based systems can achieve not only the ability to communicate over long distances, but also to aggregate information in a central server for more advanced safety functions. As a result, when a potential collision is predicted, the system supports various collision-avoidance actions such as controlling vehicles or sending warning messages to the VRU’s smartphone. Several studies have investigated V2N-based safety systems using centralized or hybrid architectures. Barmpounakis et al. [7] proposed an architecture that dynamically selects computational resources between mobile edge computing and centralized cloud-based computing for predicting hazards between vehicles and VRUs. When a potential collision is identified, the system sends warning messages to the vehicles involved through the network. Malinverno et al. [8] proposed an architecture where vehicles and pedestrians send safety messages via cellular communication to a central server, which then transmits warning messages upon collision detection. Teixeira et al. [9] developed a framework that aggregates information transmitted from vehicles, VRUs, and roadside sensors onto a single platform to detect collisions. This framework has undergone testing in real-world environments, including a comparative analysis of the performance of edge and centralized cloud architectures. Wies et al. [10] implemented a prototype that detects collisions at a central server using messages transmitted from vehicles and cyclists and then sends warning messages. This prototype has also been tested in real-world environments. These studies demonstrate the feasibility and effectiveness of V2N-based architecture for collision detection and warning.

As summarized above, V2N-based safety systems for VRUs that aggregate and integrate information from multiple sources have been investigated in many studies, and their feasibility and effectiveness have been demonstrated for collision detection or warning. However, most existing studies focus on functions in limited areas such as single intersections, and do not explicitly consider system behavior for the entire road network. To realize the widespread deployment of such systems, it is necessary to consider their effectiveness over broader road networks with limited information sources. Therefore, this study aims to predict VRU movement throughout the entire community road network under limited information availability. This extends the application of V2N-based safety systems to road networks.

### 2.2. Stochastic Prediction Method for Collision Prevention

To prevent collisions between traffic participants, safety systems perform a two-step prediction: predicting the movement of traffic participants based on integrated information from multiple sources, and predicting the collision based on their positions. The important point is to perform stochastic estimation according to the observation uncertainty and the variation in traffic participants’ movements. By using a continuous collision metric based on stochastic approaches, the system can perform flexible and adaptive collision prevention instead of relying on a simple deterministic evaluation. In the following part, existing methods for stochastic estimation are organized from three perspectives: position prediction, information integration, and collision prediction.

As for methods of position prediction, the Kalman Filter and its variations, such as the Extended Kalman Filter (EKF), Unscented Kalman Filter (UKF) [11], and Cubature Kalman Filter [12], are commonly used. These methods represent the uncertainty of observation information and estimated states as Gaussian distributions. In parallel, data-driven approaches have been investigated to predict more complex behaviors, such as lane changes, turning at intersections, and interaction between vehicles and VRUs. For instance, Chen et al. [13] analyzed lane-changing behavior of vehicles using high-resolution trajectory data collected by drones. Kawasaki et al. [14] proposed a method to improve motion estimation of vehicles at intersections by collecting time-series data of vehicle velocity at intersections. Zhang et al. [15] developed models for pedestrian crosswalk crossing prediction and locomotion prediction using data collected by an unmanned aerial vehicle. Li et al. [16] developed a model for predicting pedestrian and vehicle movements at intersections using LiDAR and camera data collected at the intersection. Tiger and Heintz [17] investigated motion pattern recognition in urban road networks using Gaussian Process models trained on vehicle GNSS trajectory data. While these data-driven methods can capture complex motion patterns, they require large amounts of data and often lack generalization capabilities across different environments. More recently, deep learning based methods have been introduced to estimate positions and associated uncertainty. Zhao et al. [18] developed a model that outputs the predicted position of pedestrians and their confidence intervals using a Gated Recurrent Unit and utilizes it for collision risk evaluation with vehicles. Nayak et al. [19] investigated a method for estimating pedestrian positions and their uncertainty using deep learning models with Monte Carlo dropout. Furthermore, Nayak et al. [20] proposed a method for probabilistic trajectory prediction of occluded targets. They trained a Long-Short-Term-Memory-based model and applied Monte Carlo dropout to obtain estimation uncertainty. These methods output estimations with associated uncertainty, while general deep learning methods output only estimations. Although such deep learning methods tend to provide improved generalization compared with classical data-driven approaches, these methods still have challenges in terms of computational efficiency and interpretability for use in safety systems. Therefore, this study uses EKF for movement estimation of traffic participants.

After the positions of two traffic participants are estimated as probability distributions, a collision metric between them can be calculated as a continuous value based on these distributions. Many existing methods for calculating collision metrics use Monte Carlo sampling. This approach first samples the states of both traffic participants with *N* samples from the predicted distributions. It then computes the proportion of samples that result in collisions as the evaluation metric. For example, Lambert et al. [21] proposed an algorithm for calculating collision probability using Monte Carlo sampling when time-series positions are estimated as normal distributions. Houénou et al. [22] proposed an approach to estimate collision probability over time from the predicted position distributions at each time step using Monte Carlo sampling. Campos et al. [23] proposed a method that predicts the positions of two vehicles by UKF and performs control interventions based on the calculated collision probability. Tao et al. [24] developed a method that predicts the positions of multiple vehicles using square-root UKF and estimates collision probability. They tested the proposed method in real environments. Furthermore, Gao et al. [25] proposed a framework for position prediction that incorporates drivers’ evasive behaviors into EKF and estimates collision probability using Monte Carlo sampling. As summarized above, Monte Carlo sampling is a frequently used and generally applicable method. However, it has challenges in terms of computational cost. This is because *N* sampling calculations are required for each traffic participant at each time step of collision prediction. Based on the above considerations, this study projects the estimated two-dimensional position distribution onto a one-dimensional direction along the road for collision metric calculation. Because the integral of a one-dimensional Gaussian distribution can be computed at low computational cost, this approach is effective for maintaining performance of the system for multiple agents.

### 2.3. Cooperative Prediction Using Multiple Information Sources

When integrating diverse information from multiple sources, one important point is the consideration of information correlation. In real systems, information is not always independent and can be correlated. If information integration is performed naively without considering this correlation, the problem of “data incest” arises, and the estimation uncertainty is underestimated. For such a problem, Covariance Intersection (CI) [26] and Split Covariance Intersection [27] are generally used in existing research on cooperative localization for vehicles. It has been shown that information integrated using these methods is always “conservative” with respect to the true uncertainty and does not underestimate it. Li et al. [28] proposed a cooperative localization method for multiple vehicles under a highway scenario, where vehicles can mutually observe each other. The proposed method improves the accuracy of vehicles’ localization by cooperative integration of state estimation based on Split Covariance Intersection. Héry et al. proposed a method [29] to cooperatively improve localization accuracy using CI in a situation where two vehicles observe each other. Subsequently, they proposed a method [30] for cooperative localization in platooning with a larger number of vehicles. Furthermore, Cai et al. [31] proposed a cooperative localization method for multiple vehicles based on Cubature Kalman Filter and Split Covariance Intersection.

The above studies are similar to our study because they consider the uncertainty of the vehicle’s self-localization and the data incest problem. However, they assume a situation where multiple vehicles continuously observe each other on main roads. On community roads, traffic density is low, and the number of roadside sensors is also limited. Therefore, unlike existing research where periodic observation information is assumed to be available, it is necessary to continuously predict VRU movement using sparse information.

### 2.4. Position Estimation Using Road Geometry

Because traffic participants moving on roads are constrained by road geometry, information on road geometry is useful for estimating their movement. In particular, when available sensor observations are sparse, the system needs to incorporate this information for continuous estimation after a brief observation opportunity. Therefore, this study utilizes road geometry obtained from map information. The term “map” covers a wide range of representations, from standard maps for ordinary car navigation to High-Definition (HD) maps for autonomous driving. Ideally, the use of HD maps is desirable because they provide rich geometric information with high accuracy. However, due to the high cost of map creation and maintenance, the availability of HD maps on community roads is limited [32]. In contrast, car navigation maps are already available for most road networks. For example, in Japan, digital road maps have been developed [33] to represent road networks as nodes corresponding to intersections and links connecting them. However, these maps primarily focus on topological representation and lack detailed geometric features for microscopic motion estimation. To bridge the gap between these two map representations, several studies have investigated map construction with intermediate information levels. For example, some approaches digitize scanned map documents to extract road geometry [34], while others enhance navigation maps using vehicle trajectory data [35,36]. In summary, map representations vary widely, and there exists a trade-off between the quality of the information and the availability. In this study, we assume the availability of map data with an intermediate level of detail that consists of intersections, roads, and road widths.

Next, we describe methods for incorporating map information into position estimation. The most direct approach is to use road coordinate systems such as the Frenet frame [37]. This approach makes it possible to express movements simply, even when the road geometry contains complex curves. However, when integrating information from various devices at multiple locations, the calculation of the coordinate transformation and uncertainty propagation becomes complex. Therefore, in this research, we express information using the Cartesian coordinate system. With Cartesian coordinate representation, road shape constraints can be incorporated into the filter in several ways. One way of actual implementation is to impose an equality constraint on the filter [38]. However, this method strictly constrains the position to the center of the road and makes it impossible to consider the position in the lateral direction. As methods to express shape constraints in a more flexible way than such strict equality constraints, two approaches are proposed: directional process noise and pseudo-measurements [39]. This incorporates prior knowledge that the target’s position does not largely vary in the lateral direction of the roads. The former adjusts uncertainty by setting the process noise values in the road’s longitudinal and lateral directions to large and small values, respectively [40,41]. On the other hand, the latter approach uniformly incorporates road constraint information by treating road constraints as observations from pseudo-sensors [42,43]. When comparing these two approaches, the former assumes straight roads, while the latter can handle curved roads as long as the road shape can be expressed by equations of *x* and *y*. For example, Zhang et al. [44] formulated road shapes as quadratic curves and provided pseudo-measurements to a filtering algorithm for position estimation. Therefore, this study adopts the latter approach in terms of generality.

Road geometry can also complicate position estimation, and route branching at intersections is a main example of this issue. When an intersection is connected to multiple roads, the system needs to assume multiple route branches for a traffic participant. Existing approaches continue estimation by generating multiple modes for each corresponding route. Kirubarajan et al. [40] proposed a method for estimating a vehicle’s position by using the Variable Structure Interacting Multiple Model. Their method continues estimation by temporarily generating multiple modes corresponding to each adjacent road at intersections. Zhang et al. [44] incorporate road shape constraints into the Variable Structure Interacting Multiple Model as pseudo-measurements for the vehicle’s position estimation. Krishanth et al. [41] developed a position prediction method that can handle route branching during unobserved periods, assuming situations where the target is not observed for a long time. Near intersections, they handle route branching by generating hypotheses for each road connected to the intersection and eliminating hypotheses when the target is observed. The method used by Krishanth et al. is the most similar to our study. Dealing with long unobservable periods is a common challenge on community roads, where available sensor information is spatially sparse. On the other hand, they assume the observation information from a single satellite or aircraft. In actual community road environments, it is necessary to cooperatively utilize observation information from multiple roadside sensors and vehicles. To overcome this limitation, this study proposes a framework that integrates road geometry information into observation information from spatially distributed information sources.

## 3. Framework for VRU Movement Prediction with Route Branching

### 3.1. Framework Overview

**Figure 4:**
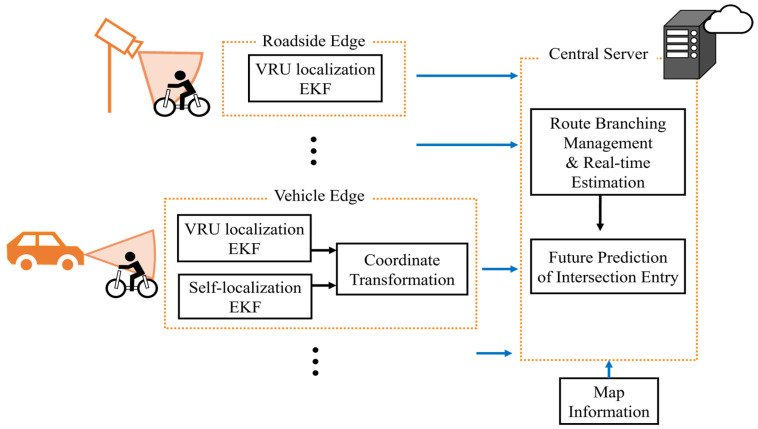
Overview of proposed framework.

Figure 4 illustrates the overview of the proposed framework. The proposed framework mainly consists of three components: the Vehicle Edges, the Roadside Edges, and the Central Server. Each Vehicle Edge and Roadside Edge possesses its own computational unit and sensors. Each Edge estimates the VRU’s movement by EKF using data from equipped sensors. The Central Server receives these estimation results from multiple Edges via a wireless network and fuses them into a unified estimation by CI. In this study, a single VRU is assumed to exist in the environment. Accordingly, data association among multiple VRUs is not considered, and all estimations from Edges are assumed to correspond to the same VRU. Then, the Central Server incorporates the map information to explicitly consider the effect of road geometry on VRU movement. Specifically, it incorporates constraints of road shapes by pseudo-observations and handles route branching at intersections. In addition to this state estimation, the Central Server predicts the future entry of VRUs into intersections, which will be used for collision avoidance.

### 3.2. Formulation of State Variables and Extended Kalman Filter

In this study, we design movement state x and its error covariance matrix P as follows:

(1) $$x={\left[x,y,\theta ,v\right]}^{T}$$

(2) $$P=\left[\begin{array}{cccc} \sigma_{xx}^{2} & \sigma_{xy} & \sigma_{x\theta } & \sigma_{xv}\\ \sigma_{xy} & \sigma_{yy}^{2} & \sigma_{y\theta } & \sigma_{yv}\\ \sigma_{x\theta } & \sigma_{y\theta } & \sigma_{\theta \theta }^{2} & \sigma_{\theta v}\\ \sigma_{xv} & \sigma_{yv} & \sigma_{\theta v} & \sigma_{vv}^{2} \end{array}\right]$$

where x and y denote positions in a Cartesian coordinate system, θ the yaw angle, and v the velocity. In the proposed framework, each component estimates the movement of traffic participants and represents it as an estimated state $\hat{x}$ and estimated error covariance matrix $\hat{P}$. In addition, these estimates are assumed to follow a Gaussian distribution; the estimated distribution is denoted as $N(\hat{x},\hat{P})$. Based on movement state x, control input u is defined as follows:

(3) $$u={\left[\omega ,\;a\right]}^{T}$$

where ω denotes the yawing rate and a the acceleration. In addition, x and u at time step t are expressed as follows:

(4) $$x_{t}={\left[x_{t},\;y_{t},\theta_{t},v_{t}\right]}^{T}$$

(5) $$u_{t}={\left[\omega_{t},\;a_{t}\right]}^{T}$$

Based on the above state and input variables, EKF conducts motion estimation to obtain estimated state ${\hat{x}}_{t}$ and covariance ${\hat{P}}_{t}$. Specifically, EKF consists of the following two steps:

1. Prediction step, which predicts the state and its covariance by a nonlinear motion model and the control input.
2. Update step, which updates the state and its covariance by observation information.

The detailed formulation of EKF and the specific values of parameters are provided in Appendix A.

### 3.3. Roadside Edge and Vehicle Edge

**Figure 5:**
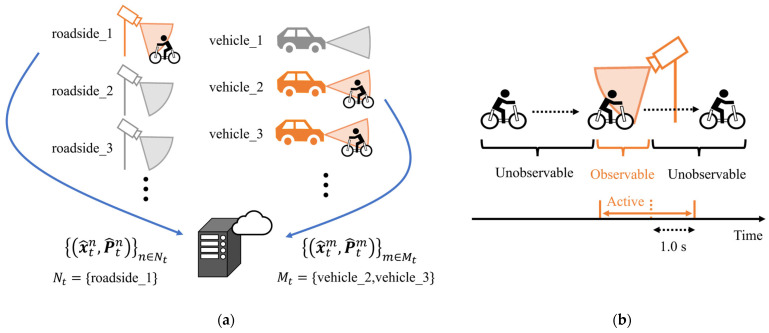
(**a**) Overview of the information provision from the multiple Edges to the Central Server. (**b**) Process of activating and deactivating information provision in Edges.

At the Roadside Edges and the Vehicle Edges, the movements of traffic participants are estimated by the EKF using information from roadside sensors and vehicles’ on-board sensors, respectively. Figure 5a illustrates the overview of the information provision from the multiple Edges to the Central Server. In the framework, multiple Roadside Edges and Vehicle Edges are uniquely identified by Edge labels. Among these Edges, not all of them always provide estimation results, and only the Edges that observe the VRU are activated for information provision. Specifically, Figure 5b illustrates the process of activating and deactivating provision in Edges. At each Edge, state estimation is performed using the EKF while the VRU exists within the observation range of the corresponding sensor. When the VRU exits the observation range and sensor observations become unavailable, the estimation is continued by repeating only the EKF’s prediction step for a duration of 1.0 s. After that, the estimation is terminated and deactivated. As a result, at a given time *t*, sets of estimation results ${\left\{\left({\hat{x}}_{t}^{n},{\hat{P}}_{t}^{n}\right)\right\}}_{n\in N_{t}}$ and ${\left\{\left({\hat{x}}_{t}^{m},{\hat{P}}_{t}^{m}\right)\right\}}_{m\in M_{t}}$ are obtained from the active Roadside Edges and Vehicle Edges, where $N_{t}$ and $M_{t}$ denote the label set of the active Roadside Edges and Vehicle Edges, respectively.

Next, we describe the coordinate systems used for estimation at Roadside Edges and Vehicle Edges. As for the Roadside Edges, VRU movement estimation is performed explicitly in the global coordinate system. This is because roadside sensors are assumed to be calibrated at installation and their observations are directly obtained in the global coordinate system. On the other hand, unlike roadside sensors with fixed positions, vehicles’ on-board sensors move with the vehicles. Therefore, estimation by the Vehicle Edges needs to consider the vehicle’s self-localization in the global coordinate system and the VRU localization in the vehicle’s local coordinate system. In this study, we assume the architecture investigated in our previous work [45] to obtain estimation results from Vehicle Edges. The abstracts of this architecture are provided in Appendix B. As a result, the estimation results from both Roadside Edges and Vehicle Edges are represented in the global coordinate system.

### 3.4. Central Server

#### 3.4.1. Overview of Functional Components in the Central Server

**Figure 6:**
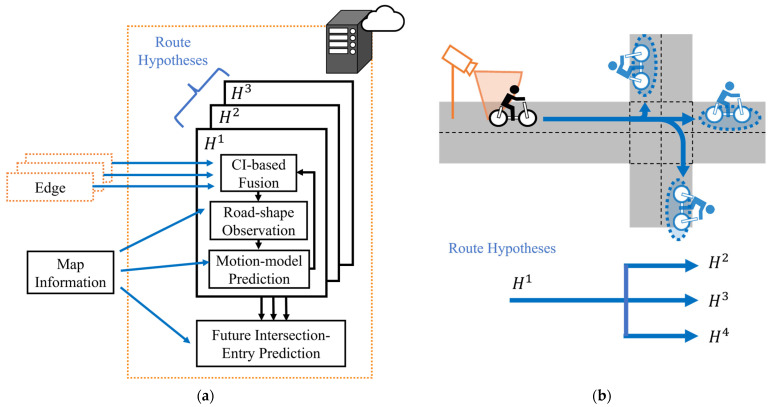
(**a**) Overview of Central Server. (**b**) Management of route branching at intersections.

Figure 6a,b illustrate the overview of the Central Server and the route branching at intersections, respectively. A distinctive feature of the Central Server is the management of multiple route hypotheses. As shown in Figure 6b, these route hypotheses are generated to continue estimation after route branching at intersections. Each hypothesis independently maintains its own estimated state and covariance matrix, and estimation is performed in parallel across all hypotheses over time. In this process, estimation results from the Roadside Edges and Vehicle Edges are associated with the corresponding route hypotheses and fused by CI. Then, the Central Server incorporates map information into each estimation using pseudo-observations and performs predictions using the prediction step of the EKF. In addition to this estimation, the Central Server aggregates all route hypotheses and predicts the entry of the VRU into intersections at a future time.

#### 3.4.2. Generation of Route Hypotheses

On road networks with intersections, the future route of a traffic participant after entering an intersection cannot be uniquely determined. If the prediction assumes only a single route, the estimation rapidly degrades once the assumed route differs from the actual one. To address this issue, the Central Server explicitly represents route branching at intersections by maintaining multiple route hypotheses. Let $L_{t}$ denote an index set of active route hypotheses at time step *t* and let hypotheses be indexed by i (i=1,2,…). Each hypothesis consists of its estimated state, its covariance matrix, and its weight, as follows:

(6) $$H_{t}^{i}=\left\{{\hat{x}}_{t}^{i},{\hat{P}}_{t}^{i},{\hat{w}}_{t}^{i}\right\}$$

where ${\hat{w}}_{t}^{i}\in [0,1]$ denotes the hypothesis weights. The weights satisfy the following constraint:

(7) $$\sum_{i{\in L}_{t}}{\hat{w}}_{t}^{i}=1.$$

Here, when a hypothesis is removed, the weights of the remaining hypotheses are normalized so that their sum meets Equation (7) while preserving their relative proportions.

**Figure 7:**
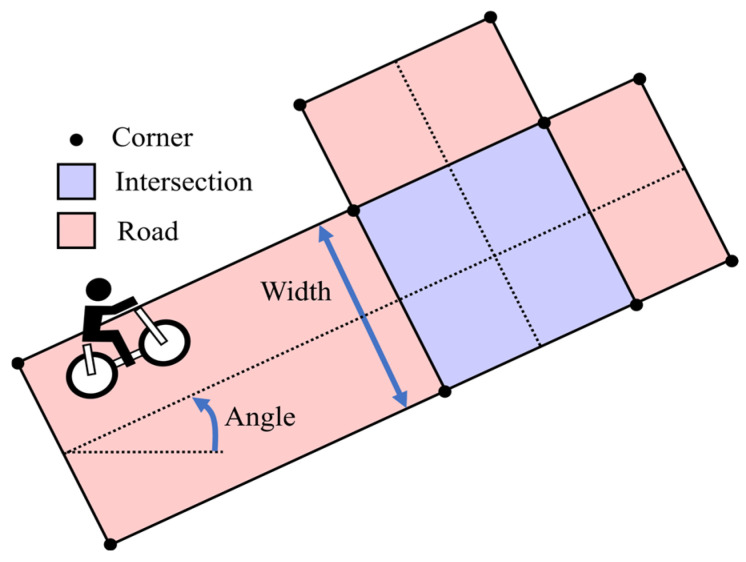
Map information assumed in this study.

**Figure 8:**
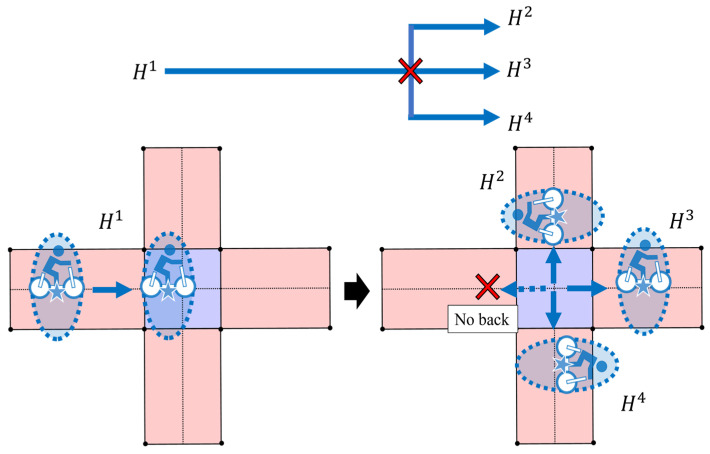
Process of hypothesis generation at intersections.

Next, we explain the generation of route hypotheses at an intersection. Figure 7 illustrates the available map information assumed in this study. The map information includes polygon-based representations of roads and intersections. In this study, all roads are assumed to be straight segments with a constant width. Therefore, we assume that information on the width and the orientation of road segments is available for movement estimation. In addition, Figure 8 illustrates the process of hypothesis generation at intersections. The route hypotheses are generated when the following two conditions are simultaneously satisfied:

1. First, the estimated position distribution overlaps with an intersection region. Here, the position distribution is represented by a 95% confidence ellipse.
2. Second, the estimated position becomes closest to the center of the intersection. To be more precise, when the distance to the intersection center begins to increase at the next step, the current step is regarded as the point of minimum distance.

When these conditions are met, route hypotheses are generated based on the set of roads connected to the intersection, except for the road on which the traffic participant was located before entering the intersection. In other words, a route that goes back to the same road is not considered.

In the following part, we assume that a set of child hypotheses $\left\{H_{child}\right\}$ is generated from a parent hypothesis $H_{parent}$. First, the estimated state ${\hat{x}}_{child}$ is updated from ${\hat{x}}_{parent}$. Specifically, the orientation difference ∆θ is calculated between the current state and the next road, and the correction is applied to the state:

(8) $${\hat{x}}_{child}={\hat{x}}_{parent}+{\left[\begin{array}{cccc} 0 & 0 & ∆\theta & 0 \end{array}\right]}^{T}$$

Next, the estimated covariance ${\hat{P}}_{child}$ is updated from ${\hat{P}}_{parent}$. Here, the position-related elements of the covariance matrix are rotated as follows:

(9) $${\hat{P}}_{child}=\left[\begin{array}{cccc} \cos\left(∆\theta \right) & -\sin\left(∆\theta \right) & 0 & 0\\ \sin\left(∆\theta \right) & \cos\left(∆\theta \right) & 0 & 0\\ 0 & 0 & 1 & 0\\ 0 & 0 & 0 & 1 \end{array}\right]{\hat{P}}_{parent}{\left[\begin{array}{cccc} \cos\left(∆\theta \right) & -\sin\left(∆\theta \right) & 0 & 0\\ \sin\left(∆\theta \right) & \cos\left(∆\theta \right) & 0 & 0\\ 0 & 0 & 1 & 0\\ 0 & 0 & 0 & 1 \end{array}\right]}^{T}$$

Then, the hypothesis weight ${\hat{w}}_{child}$ is updated from ${\hat{w}}_{parent}$. Let $N_{child}$ denote the number of newly generated child hypotheses $\left\{H_{child}\right\}$. The weight of each child hypothesis is initialized by equally dividing the weight of the parent hypothesis, as follows:

(10) $${\hat{w}}_{child}=\frac{{\hat{w}}_{parent}}{N_{child}}\;.$$

Finally, $\left\{H_{child}\right\}$ are added to the active hypothesis set and $H_{parent}$ is removed from the active hypothesis set.

#### 3.4.3. Integration of Estimations from Edges Using Covariance Intersection

Although each hypothesis performs state estimation in a similar manner to a general EKF, the ordinary update step is replaced with CI when fusing multiple Edge estimations. This is because identical motion models are used at all Edges, and the cross-correlations among estimation results cannot be accurately identified. In addition to the correlation among multiple Edges, in the proposed framework, estimation results provided by each Edge are not statistically independent across time. This is because they are sequential EKF outputs that accumulate past information. Under such conditions, the naive fusion method that assumes independent information results in an underestimation of uncertainty. Therefore, we introduce CI to fuse the estimation results in a consistent manner even when error correlations are unknown. As for the effect of the CI fusion, we describe comparative analysis of naive fusion and CI fusion in Appendix C.

**Figure 9:**
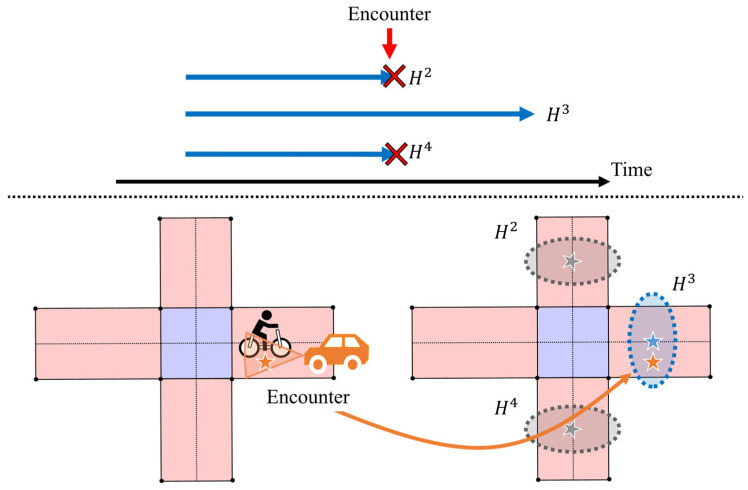
Association process between provided estimation and hypotheses, and removal of incompatible hypotheses.

First, we explain the association between estimation results from the Edges and the route hypotheses. Figure 9 illustrates the overview of the association process and the removal of incompatible hypotheses. Let the set of estimation results from Vehicle Edges and Roadside Edges at time step *t* be ${\left\{\left({\hat{x}}_{t}^{k},{\hat{P}}_{t}^{k}\right)\right\}}_{k\in N_{t}\cup M_{t}}$. In the association process, the Mahalanobis distance is calculated for all combinations of the provided estimation $\left({\hat{x}}_{t}^{k},{\hat{P}}_{t}^{k}\right)$ and hypothesis’s estimation ${(\hat{x}}_{t}^{i},{\hat{P}}_{t}^{i})$, as follows:

(11) $$d_{t}^{i,k}=\sqrt{{\left({\hat{x}}_{t}^{k}-{\hat{x}}_{t}^{i}\right)}^{T}{\left({\hat{P}}_{t}^{k}+{\hat{P}}_{t}^{i}\right)}^{-1}\left({\hat{x}}_{t}^{k}-{\hat{x}}_{t}^{i}\right)}$$

Then, if the squared Mahalanobis distance ${\left(d_{t}^{i,k}\right)}^{2}$ is smaller than a predefined threshold, the provided estimation $\left({\hat{x}}_{t}^{k},{\hat{P}}_{t}^{k}\right)$ and hypothesis’s estimation ${(\hat{x}}_{t}^{i},{\hat{P}}_{t}^{i})$ are regarded as associated, and they are fused in the following CI fusion step. Specifically, we define a 95% confidence region as the gating threshold in this study. Furthermore, hypotheses that are not associated with any provided estimation are removed from the active hypothesis set.

Next, we explain the process of the CI fusion. Let $\left({\hat{x}}_{t}^{i,prior},{\hat{P}}_{t}^{i,prior}\right)$ be the prior estimation of $H^{i}$ at time step *t*, and let $\left({\hat{x}}_{t}^{k},{\hat{P}}_{t}^{k}\right)$ be an external estimation result from a Vehicle Edge or a Roadside Edge. The CI fusion combines these two estimations to obtain a posterior estimation $\left({\hat{x}}_{t}^{i,post},{\hat{P}}_{t}^{i,post}\right)$, as follows:

(12) $${\hat{P}}_{t}^{i,post}={\left\{\omega_{t}^{i,k}{\left({\hat{P}}_{t}^{i,prior}\right)}^{-1}+\left(1-\omega_{t}^{i,k}\right){\left({\hat{P}}_{t}^{k}\right)}^{-1}\right\}}^{-1}$$

(13) $$\begin{array}{c} {\hat{x}}_{t}^{i,post} \end{array}={\hat{P}}_{t}^{i,post}\left\{\omega_{t}^{i,k}{\left({\hat{P}}_{t}^{i,prior}\right)}^{-1}{\hat{x}}_{t}^{i,prior}+\left(1-\omega_{t}^{i,k}\right){\left({\hat{P}}_{t}^{k}\right)}^{-1}{\hat{x}}_{t}^{k}\right\}$$

where $\omega_{t}^{i,k}\in [0,1]$ is a weighting parameter which is selected to reduce a metric of the fused covariance ${\hat{P}}_{t}^{i,post}$. In this study, we select ω to reduce $tr({\hat{P}}_{t}^{i,post})$, which corresponds to the sum of the variances of the estimated state. Specifically, we use Brent’s method implemented in SciPy [46].

#### 3.4.4. Pseudo-Observations of Road Shape

Because the movement of traffic participants on roads is constrained by road shapes, the Central Server incorporates road shape information as pseudo-observations to improve the estimation of the VRU’s movement. Let $\theta_{r}$ denote the orientation of a given road segment *r*, and $p_{r}^{rep}={\left[x_{r}^{rep},y_{r}^{rep}\right]}^{T}$ the representative position on the road in the global coordinate system. This road segment is expressed by the line equation of position (x,y) as follows:

(14) $$-\sin\left(\theta_{r}\right)x+\cos\left(\theta_{r}\right)y=c_{r}^{rep}$$

(15) $$c_{r}^{rep}=\left[\begin{array}{c} -\sin\left(\theta_{r}\right)\\ \cos\left(\theta_{r}\right) \end{array}\right]\cdot \;p_{r}^{rep}$$

Here, $p_{r}^{rep}$ can be defined as a fixed position on the road, such as the one on the centerline. However, repeatedly applying pseudo-observations with a fixed representative position leads the estimated lateral position to converge toward the fixed position, which causes an unfavorable bias in estimation. Therefore, in this study, we use the current estimated position ${\left[{\hat{x}}_{t}^{i},{\hat{y}}_{t}^{i}\right]}^{T}$ as $p_{r}^{rep}$ at each time step.

Assuming that the VRU moves along a straight road segment indicates that its position is constrained by Equations (14) and (15), pseudo-observations incorporate this constraint into the state estimation in a soft manner with uncertainty. Specifically, when the VRU is located on road segment *r*, the update step is performed using the following observation matrix ***H***, pseudo-observation ***z***, and observation noise covariance ***R***:

(16) $$H=\left[\begin{array}{cccc} -\sin\left(\theta_{r}\right) & \cos\left(\theta_{r}\right) & 0 & 0 \end{array}\right]$$

(17) $$z=\left[c_{r}^{rep}\right]$$

(18) $$R=\left[{\left(\sigma_{r}^{width}\right)}^{2}\right]$$

where $\sigma_{r}^{width}$ denotes the uncertainty of the VRU’s lateral position on the road segment. This parameter represents both the accuracy of the map information and the variability of the VRU’s lateral position within the road width.

In addition, a constraint on the road orientation is incorporated by a pseudo-observation, as follows:

(19) $$H=\left[\begin{array}{cccc} 0 & 0 & 1 & 0 \end{array}\right]$$

(20) $$z=\left\{\begin{array}{c} \left[\theta_{r}\right],\;if\;hypothesis\;direction\;is\;consistent\;with\;road\;orientation\\ \left[{-\theta }_{r}\right],\;otherwise\;\;\;\;\;\;\;\;\;\;\;\;\;\;\;\;\;\;\;\;\;\;\;\;\;\;\;\;\;\;\;\;\;\;\;\;\;\;\;\;\;\;\;\;\;\;\;\;\;\;\;\;\;\;\;\;\;\;\;\;\;\;\;\;\;\;\;\;\;\;\;\;\;\;\;\;\;\;\;\;\;\;\;\;\;\;\;\;\;\;\;\;\;\;\;\; \end{array}\right.$$

(21) $$R=\left[{\left(\sigma_{r}^{ori}\right)}^{2}\right]$$

where $\sigma_{r}^{ori}$ denotes the uncertainty of the VRU’s orientation on the road segment. Similar to $\sigma_{r}^{width}$, this parameter represents both the accuracy of the map information and the variability of the VRU’s orientation along the road segment. The specific value of $\sigma_{r}^{width}$ and $\sigma_{r}^{ori}$ are described in Appendix A.

#### 3.4.5. Future Prediction and Calculation of Intersection-Entry Certainty

**Figure 10:**
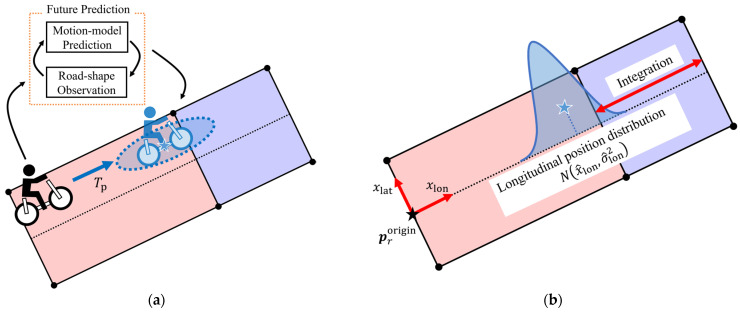
Overview of intersection-entry prediction: (**a**) future prediction; (**b**) projection onto road direction and integration over intersection segment.

In addition to state estimation using multiple route hypotheses, the Central Server predicts whether the VRU will enter an intersection after a predefined prediction horizon. Figure 10 illustrates the overview of the intersection-entry prediction. The prediction consists of three steps: future prediction for each hypothesis, projection onto the road direction, and integration over the intersection segment. Each step is described below.

First, we describe the future prediction. In the estimation, a route hypothesis $H_{i}$ maintains an estimated state and its covariance ${(\hat{x}}_{t}^{i},{\hat{P}}_{t}^{i})$. The future prediction uses these values as the initial condition to estimate the state after the prediction horizon. Here, this future prediction uses the EKF’s prediction step by the motion model and pseudo-observations of the road shape information (Equations (16)–(21)). This process is applied independently to all route hypotheses at each time step. Let $T_{p}$ denote the future prediction horizon, and ${\hat{x}}_{t+T_{p}}^{i}\;and\;{\hat{P}}_{t+T_{p}}^{i}$ the predicted future state and covariance matrix for hypothesis $H_{i}$. Through the future prediction, a set of future state and covariance matrix ${\left\{\left({\hat{x}}_{t+T_{p}}^{i},{\hat{P}}_{t+T_{p}}^{i}\right)\right\}}_{i\in L_{t}}\;$ is obtained at time step *t*.

Next, we describe the projection onto the road direction. For the prediction of VRUs’ entrance into intersections, this study does not directly use the two-dimensional position distribution. Instead, the position distribution is projected onto a one-dimensional coordinate aligned with the road direction. Let ${\hat{x}}_{pos}={\left[\hat{x},\hat{y}\right]}^{T}$ denote the position-related elements of $\hat{x}$, and ${\hat{P}}_{pos}$ the position-related submatrix of $\hat{P}$. The position and covariance matrix in the road-aligned coordinate system is calculated from ${\hat{x}}_{pos}$ and ${\hat{P}}_{pos}$, as follows:

(22) $$\left[\begin{array}{c} {\hat{x}}_{lon}\\ {\hat{x}}_{lat} \end{array}\right]=\left[\begin{array}{cc} \cos\left({-\theta }_{r}\right) & -\sin\left({-\theta }_{r}\right)\\ \sin\left({-\theta }_{r}\right) & \cos\left({-\theta }_{r}\right) \end{array}\right]({\hat{x}}_{pos}-p_{r}^{origin})$$

(23) $$\left[\begin{array}{cc} {\hat{\sigma }}_{lon}^{2} & {\hat{\sigma }}_{lon,lat}\\ {\hat{\sigma }}_{lon,lat} & {\hat{\sigma }}_{lat}^{2} \end{array}\right]=\left[\begin{array}{cc} \cos\left({-\theta }_{r}\right) & -\sin\left({-\theta }_{r}\right)\\ \sin\left({-\theta }_{r}\right) & \cos\left({-\theta }_{r}\right) \end{array}\right]{\hat{P}}_{pos}{\left[\begin{array}{cc} \cos\left({-\theta }_{r}\right) & -\sin\left({-\theta }_{r}\right)\\ \sin\left({-\theta }_{r}\right) & \cos\left({-\theta }_{r}\right) \end{array}\right]}^{T}$$

where ${\hat{x}}_{lon}$ and ${\hat{x}}_{lat}$ denote longitudinal and lateral positions along the road, respectively. In addition, $\theta_{r}$ denotes the orientation and $p_{r}^{origin}$ the origin position of road segment *r* in the global coordinate system. Among these positions, the longitudinal component ${\hat{x}}_{lon}$ and its variance ${\hat{\sigma }}_{lon}^{2}$ are extracted and used as the one-dimensional position distribution $N\left({\hat{x}}_{lon},{\hat{\sigma }}_{lon}^{2}\right).$ In Figure 10b, the blue peak represents this one-dimensional distribution projected along the road direction.

Finally, we describe the integration of the projected future position over the intersection region. As shown in Figure 10b, the intersection region is defined as a segment along the road direction. By integrating the one-dimensional position distribution over this interval, we obtain the value defined as the certainty of the VRU entering the intersection, as follows:

(24) $$f\left(x_{lon}\right)=\frac{1}{\sqrt{2\pi {\hat{\sigma }}_{lon}^{2}}}\exp\left\{-\frac{1}{2{\hat{\sigma }}_{lon}}{\left(x_{lon}-{\hat{x}}_{lon}\right)}^{2}\right\}$$

(25) $$C=\int f\left(x_{lon}\right)dx_{lon}$$

where C denotes the intersection-entry certainty. Here, we explicitly consider the hypothesis index *i* and the time step *t.* For each hypothesis $H_{i}$, the future estimated state and covariance $\left({\hat{x}}_{t+T_{p}}^{i},{\hat{P}}_{t+T_{p}}^{i}\right)$ are projected onto the road direction, and the intersection-entry certainty $C_{t+T_{p}}^{i}$ is calculated. After that, this certainty is weighted by the hypothesis certainty ${\hat{w}}_{t}^{i}$ and we obtain ${\hat{w}}_{t}^{i}C_{t+T_{p}}^{i}$. Eventually, by summing the weighted entry certainties of the corresponding hypothesis to each intersection, the entry of the VRU into each intersection at time $t+T_{p}$ is predicted.

## 4. Simulation Design

In Section 4, we describe the simulation design to evaluate the proposed framework assuming traffic scenarios on community roads, where available observation information is sparse and route branching frequently occurs at intersections. The purpose of the simulation is to examine whether the proposed framework can continuously perform movement prediction and intersection-entry prediction with degrading uncertainty during unobservable intervals. We conduct Monte Carlo simulations where the VRU’s travel route and the locations where the VRU encounters vehicles are randomized.

### 4.1. Experimental Scenarios

**Figure 11:**
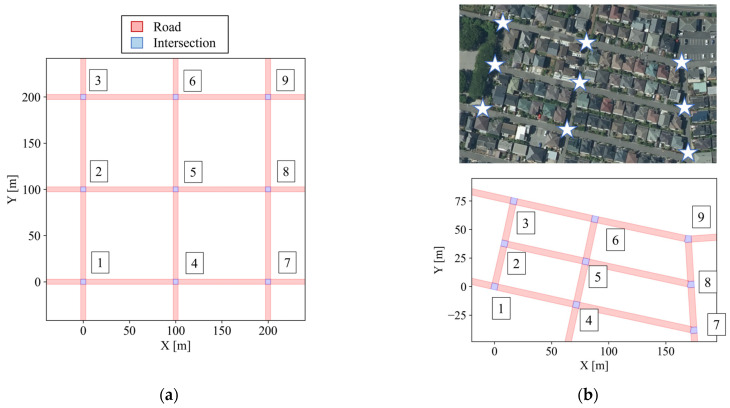
Road networks investigated in this study: (**a**) simplified grid road network with road length of 100 m; (**b**) actual network of a Japanese community road (created by editing the digital map and aerial photograph [2]).

Figure 11a,b illustrate two road networks investigated in this study. In Figure 11, red areas and blue areas indicate roads and intersections, respectively. In addition, each intersection is labeled from Intersection 1 to Intersection 9, as shown in Figure 11. The first network is a simplified grid road network with a road length of 100 m. Because community roads in residential areas are often organized in a grid-like layout, we investigate such a simplified grid road network in the simulation. In addition, the second one represents an actual network of a Japanese community road. We use this network as a case study to demonstrate the usefulness of the proposed framework under realistic road geometry.

### 4.2. Movement of Traffic Participants

**Figure 12:**
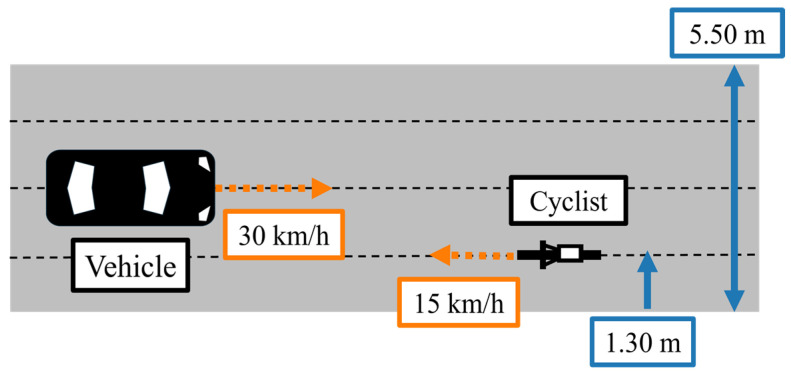
Movement of cyclist and vehicles in simulation.

Figure 12 illustrates the movement of the cyclist and vehicles in the simulations. As described in Section 3, the proposed framework assumes that a single VRU exists in the environment. In the simulations, we consider a cyclist as the VRU, and the simulated traffic scenario consists of one cyclist and multiple vehicles. As shown in Figure 12, vehicles are assumed to travel along the centerline of each road segment, while the cyclist travels on the left side of the road. This assumption reflects the Japanese traffic situation. In addition, the vehicle’s velocity is set to 30 km/h, and the cyclist’s velocity is set to 15 km/h, which are representative values for community road environments. On straight road segments, they move with constant velocity along straight lines. When turning at intersections, their trajectories follow circular arcs that connect the incoming and outgoing road segments. Furthermore, the simulation is performed with a time step of 0.1 s, and the movement of traffic participants is calculated at this interval.

### 4.3. Sensor Configurations

**Figure 13:**
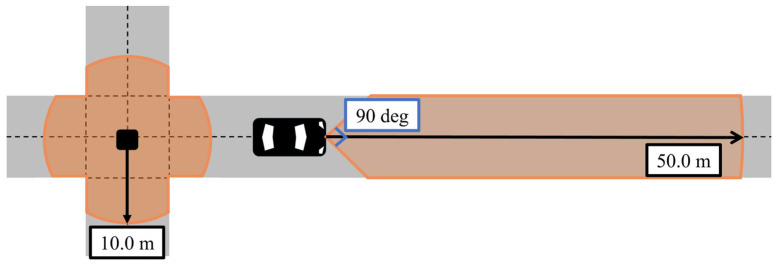
Observation coverage of vehicle’s millimeter-wave radar and roadside LiDAR.

In the simulation, sensor observations are generated by adding Gaussian noise to the ground-truth states according to sensor accuracy and then provided to each EKF in Edge. As for a vehicle’s on-board sensors, the specifications are determined by assuming widespread Advanced Driver Assistance System (ADAS)-level vehicles. Although sensor accuracy influences the estimation results to some extent, we consider that its impact is relatively limited compared to uncertainty growth and route branching during unobservable periods. Therefore, this study assumes the sensor accuracy level of widespread vehicles so that a wider range of vehicles can be utilized as Vehicle Edges within the proposed framework. In addition, we assume that the Roadside Edges are designed to observe traffic participants within intersections, and we determine that VRUs’ positions within the intersection are obtained by roadside LiDAR. Figure 13 illustrates the observation coverage of the vehicle’s millimeter-wave radar and the roadside LiDAR. The vehicle’s radar has a fan-shaped observation area with a range of 50 m and a Field of View of 90 degrees, while the roadside LiDAR has a circular area with a radius of 10 m. The detailed specifications of all sensors used in the simulation are summarized in Appendix A.

### 4.4. Configuration of Monte Carlo Simulation

**Figure 14:**
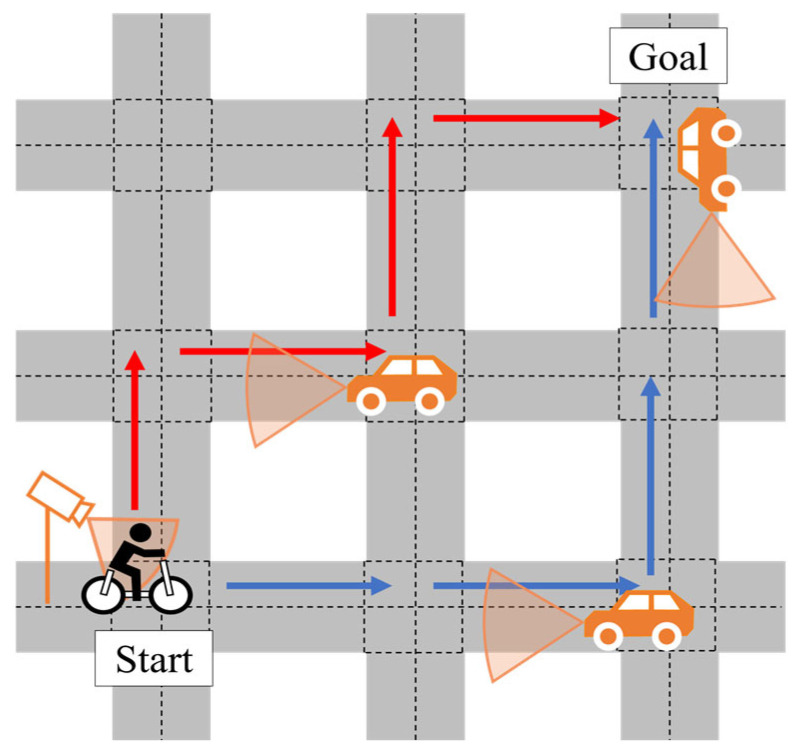
Randomized conditions in simulations.

In the Monte Carlo simulation, the number of encountering vehicles is first fixed as a predefined parameter, and multiple trials are conducted with some random conditions. The performance is evaluated by aggregating the resulting metrics. Specifically, two conditions are randomized for each trial: the travel route of the cyclist and the encountering conditions with vehicles. Figure 14 illustrates the randomized conditions in the simulations. First, we describe the route conditions. In the simulations, the start point of the cyclist’s route is fixed at the lower-left intersection of the network, and the goal point is fixed at the upper-right. The cyclist selects one of the routes between these two intersections at random, excluding routes that include backward movement or revisit of the same road segment. For example, the cyclist randomly selects one of the candidate routes, such as those illustrated by the red and blue arrows in Figure 14. Next, we describe the encountering conditions with vehicles. Under the above route conditions, the cyclist travels from the start point to the goal point through a total of four road segments. For the three segments excluding the first one, each segment can have at most one vehicle encountering event. Therefore, the number of encountering vehicles is determined as an integer between zero and three. With the fixed number of encountering vehicles, the road segments and positions where encountering occur are randomly determined. Under these randomized conditions, each trial is conducted, the cyclist’s movement is estimated by the proposed framework, and the evaluation metrics are computed. In this simulation, the number of trials is set to 100 for each number of encountering vehicles.

### 4.5. Evaluation Metrics

**Figure 15:**
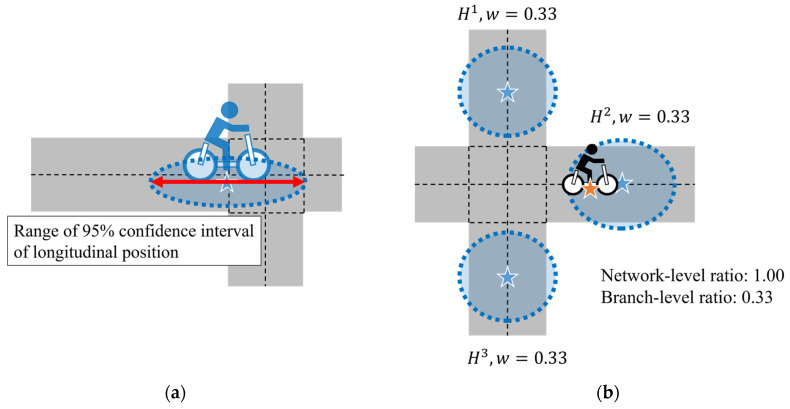
(**a**) Range of a 95% confidence interval of longitudinal position for estimated position distribution. (**b**) Calculation of two types of existence ratio within a 95% confidence interval of position.

**Figure 16:**
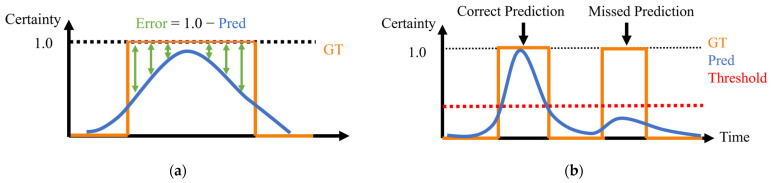
(**a**) Calculation of entry-certainty error at each intersection. (**b**) Missed entry prediction and false entry prediction.

**Figure 17:**
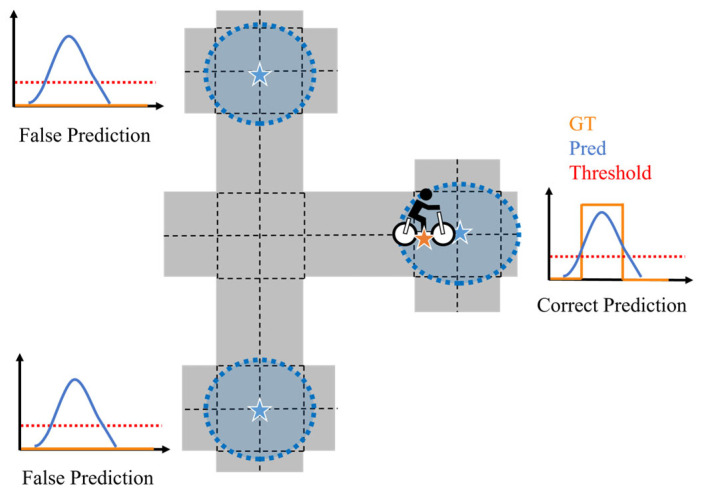
Situation where false entry prediction occurs with multiple route hypotheses.

The evaluation metrics are divided into two categories: metrics related to state estimation and metrics related to intersection-entry prediction. Figure 15, Figure 16 and Figure 17 illustrate the calculation of each metric. The details of these metrics are described in the following.

#### 4.5.1. Range of a 95% Confidence Interval of Longitudinal Position

The range of a 95% confidence interval of longitudinal position represents the position uncertainty along the direction of the cyclist’s movement. Figure 15a illustrates this range of the estimated distribution. In each trial, the range is calculated for each intersection that passed by the cyclist, except for the start intersection. Specifically, the value is evaluated at the time when the ground-truth position of the cyclist first enters each intersection.

#### 4.5.2. Average of Existence Ratio Within 95% Confidence Interval of Position

The average of the existence ratio within the 95% confidence interval of position evaluates whether the ground-truth position is contained within the estimated confidence intervals. Here, we consider two types of this ratio: network-level one and branch-level one. In the network-level ratio, the existence is counted as 1.0 if the ground-truth position is contained within the 95% confidence interval of at least one hypothesis. On the other hand, in the branch-level ratio, the existence is counted by weighting each hypothesis’s weight. In Figure 15b, the ground-truth position is contained within the confidence interval of hypothesis $H^{2}$ among the three hypotheses. Therefore, the network-level ratio becomes 1.0 and the branch-level ratio becomes 0.33, which is the weight of hypothesis $H^{2}$. In both cases, the counts are accumulated from start to end in each trial and then averaged to obtain the ratio of the trial. This definition is based on the idea that, at the road-network level, prediction can be regarded as being appropriately maintained if at least one hypothesis covers the ground-truth. On the other hand, the branch-level average ratio reflects the reliability of the prediction at a specific road segment because each hypothesis is associated with a specific route.

#### 4.5.3. Entry-Certainty Error

Entry-certainty error quantifies the difference between the predicted intersection entry certainty and the actual intersection existence of the cyclist. This metric is evaluated only when the cyclist actually exists inside an intersection, as shown in Figure 16a. At each time step, the error is defined as 1.0 minus the certainty value. Because the ideal certainty should be equal to 1.0 when the cyclist actually exists within an intersection, the error represents the deviation of the estimated certainty from this ideal value. In each trial, the range is calculated for each intersection that passed by the cyclist, except for the start intersection. Specifically, the error values at each intersection are averaged, and the resulting mean value is used as the error associated with that intersection.

#### 4.5.4. Missed Entry Prediction and False Entry Prediction

Missed entry prediction and false entry prediction metrics evaluate the intersection-entry prediction based on a predefined certainty threshold. An intersection entry is predicted when the intersection-entry certainty exceeds the threshold. A missed entry prediction is counted when the cyclist actually enters an intersection while the certainty does not exceed the threshold. In Figure 16b, the entry into the first intersection is correctly predicted because its certainty exceeds the threshold, while the entry into the second intersection is missed because its certainty is below the threshold. Furthermore, Figure 17 illustrates a situation where false entry prediction occurs. As shown in Figure 17, false prediction is counted when the certainty exceeds the threshold while the cyclist does not enter the intersection. Because these metrics depend on the selected threshold, this study evaluates four threshold values: 0.01, 0.05, 0.10, and 0.30. The design of this value for warning and intervention strategies is discussed in Section 6.

## 5. Results

### 5.1. Sample Case Analysis in a 2 × 2 Grid Network

In Section 5.1, we conduct a sample case analysis for a minimal 2 × 2 grid network to evaluate the feasibility of the proposed framework. We compare two scenarios: a scenario without any encountering vehicles and a scenario with one encountering vehicle. The purpose of this analysis is to clarify how the proposed framework behaves in state estimation and intersection-entry prediction.

**Figure 18:**
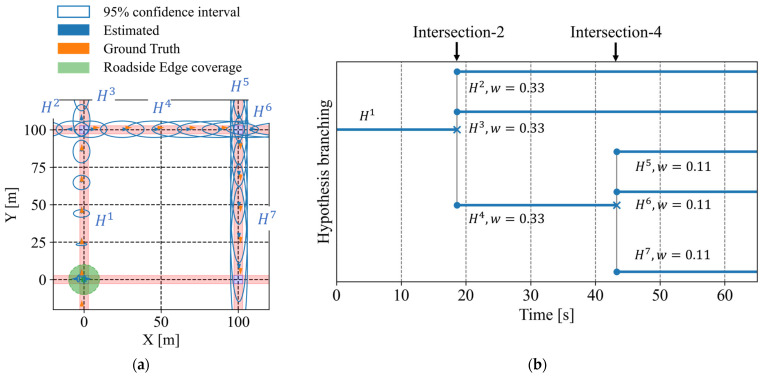
(**a**) Estimated positions according to the framework in the scenario without any encountering vehicles. (**b**) Branching of route hypotheses in the scenario without any encountering vehicles.

First, we consider a scenario where the cyclist moves through the network without any encountering vehicles. Figure 18a shows the estimated positions according to the framework. In Figure 18a, orange arrows indicate the ground-truth position and orientation of the cyclist, blue arrows the predicted position and orientation, and blue ellipses the 95% confidence intervals of the predicted position. In addition, the green area indicates the observation coverage of the roadside sensor. In this scenario, the cyclist starts from the bottom-left intersection (Intersection-1), moves to the upper-left intersection (Intersection-2), then to the upper-right intersection (Intersection-4), and finally reaches the lower-right intersection (Intersection-3). In addition, Figure 18b illustrates the branching of route hypotheses in this scenario. As shown in these figures, at each intersection, multiple hypotheses are generated to represent possible future routes. Even in the absence of observation information, the proposed framework continues prediction by considering road geometry and by managing multiple route hypotheses. However, as the cyclist continues to move through the road network and passes intersections, route hypotheses repeatedly branch. As a result, the weight assigned to each hypothesis decreases and the estimated confidence interval associated with each hypothesis expands, resulting in reduced practical usefulness of the prediction.

**Figure 19:**
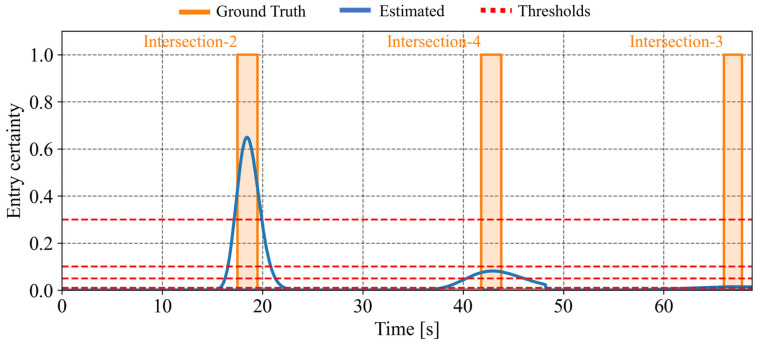
Estimated intersection-entry certainty for each intersection passed by the cyclist in the scenario without any encountering vehicles.

Figure 19 illustrates the estimated intersection-entry certainty for each intersection passed by the cyclist. In Figure 19, the orange solid line indicates the time intervals during which the cyclist actually exists within the intersection, while the blue line indicates the predicted intersection-entry certainty. In addition, the red dotted line indicates the predefined thresholds, which are set to 0.01, 0.05, 0.10, and 0.30. The certainty shown at time step *t* corresponds to a prediction conducted at ${t-T}_{p}$. In this study, the prediction horizon $T_{p}$ is set to 5.0 s. As for the first intersection, the certainty increases when the cyclist actually enters the intersection. This indicates that the framework appropriately predicts the intersection entry. In contrast, due to route branching and growing estimation uncertainty, the prediction for the second and third intersection degrades, and the certainty peak decreases. This result shows the limitation of prediction in the absence of observation information.

In addition, Table 1 summarizes the average of the existence ratio within a 95% confidence interval of position for this scenario. The network-level average existence ratio reaches 100.0%. This result indicates that the ground-truth position is always contained within at least one of the confidence intervals associated with the multiple route hypotheses. In contrast, the branch-level average existence ratio is 49.9%. While the estimation can be maintained without missing at the road-network level, the hypothesis weight is distributed across multiple route hypotheses. In other words, although a hypothesis corresponding to the true route always exists, the weight assigned to that hypothesis at each intersection continues to be dispersed. Furthermore, Table 2 shows the metrics for each intersection. The range of a 95% confidence interval of longitudinal position increases as the cyclist proceeds through intersections due to a lack of observation information. When we convert these values into Time-To-Collision (TTC) using the cyclist’s velocity, these values correspond to approximately 5 s, 14 s, and 26 s for the first, second, and third intersections, respectively. Similarly, the entry-certainty error also increases as the cyclist proceeds through intersections.

**Figure 20:**
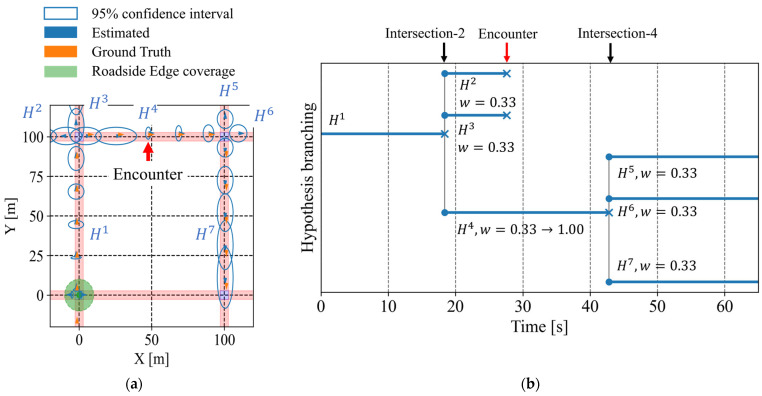
(**a**) Estimated positions according to the framework in the scenario with one encountering vehicle. (**b**) Branching of route hypotheses in the scenario with one encountering vehicle.

**Figure 21:**
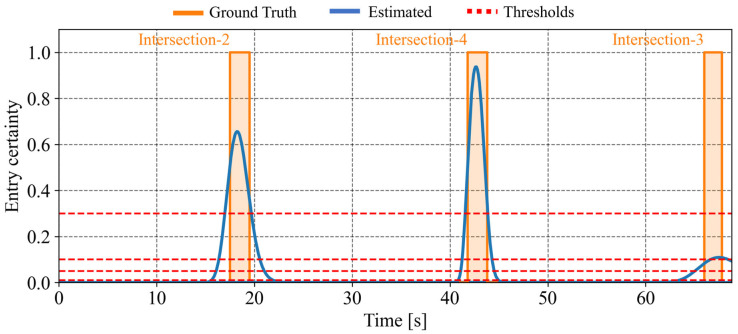
Estimated intersection-entry certainty for each intersection passed by the cyclist in the scenario with one encountering vehicle.

Next, we analyze a scenario where the cyclist and one vehicle pass each other during its movement. Figure 20a shows the estimated positions according to the framework, and Figure 20b illustrates the branching of route hypotheses in this scenario. In this scenario, the cyclist encounters a vehicle between the second and third intersections. As a result, estimated position uncertainty is reduced, as shown in Figure 20a, and inconsistent route hypotheses are removed after the encounter, as shown in Figure 20b. In addition, Figure 21 illustrates the estimated intersection-entry certainty for each intersection passed by the cyclist. The certainty associated with the third intersection improved after the encountering event. Consequently, the framework becomes capable of predicting entry into the fourth intersection. Furthermore, Table 3 and Table 4 summarize the evaluation metrics for this scenario. Compared with the no-encountering scenario, the branch-level average existence ratio within the 95% confidence interval of position shows improvement. In addition, the encounter reduces the position uncertainty and makes the range of the confidence interval at the third intersection more usable.

In summary, from the sample case analysis in the 2 × 2 grid network, the proposed framework maintains network-level estimation even with sparse observation information. Moreover, when additional observation information from vehicles becomes available, the framework improves the prediction. These results demonstrate the feasibility of the proposed framework.

### 5.2. Monte Carlo Evaluation in 3 × 3 Grid Network

Section 5.2 evaluates the proposed framework using Monte Carlo simulations in a 3 × 3 grid network. While Section 5.1 focused on the basic behavior of the framework in minimal scenarios, this section examines the validity against changes in randomized conditions and the usefulness for the availability of observation information. Specifically, we change the number of encountering vehicles from zero to three and conduct 100 trials for each number to analyze changes in the evaluation metrics.

First, Table 5 summarizes the average existence ratio within the 95% confidence interval of position. The network-level average ratio exceeds 95% under all encountering vehicle numbers. This indicates that the ground-truth position is appropriately contained within at least one of the estimated confidence intervals across all hypotheses, and this supports the validity of the proposed framework against changes in movement conditions. On the other hand, the branch-level average ratio clearly depends on the number of encountering vehicles. Without encountering vehicles, the branch-level average ratio remains at 39.48%. As the number of encountering vehicles increases, the branch-level average existence ratio increases steadily and becomes 56.19%, 69.20%, and 81.11% for one, two, and three encountering vehicles, respectively. This trend shows that additional observation information provided by encountering vehicles improves the practical usefulness of the state estimation.

**Figure 22:**
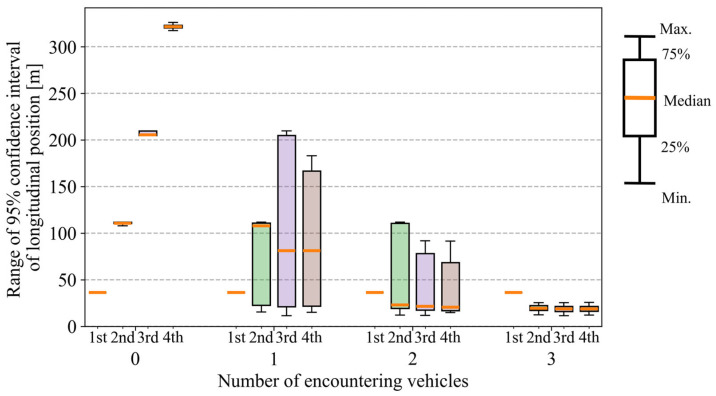
Boxplots of the range of a 95% confidence interval in longitudinal position.

**Figure 23:**
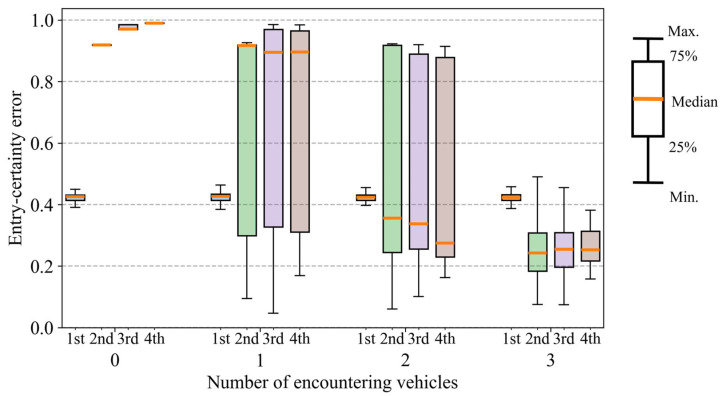
Boxplots of entry-certainty error across all trials.

Next, Figure 22 shows boxplots of the range of a 95% confidence interval of longitudinal position across all trials. In Figure 22, the horizontal axis represents the number of encountering vehicles (zero to three). For each encounter condition, boxplots are shown separately for the first to fourth intersections along the VRU’s route. Each boxplot summarizes the distribution of the confidence-interval range evaluated at the time when the VRU first enters the corresponding intersection. As shown in Figure 22, the range for each intersection decreases as the number of encountering vehicles increases. In addition, as for the variance of the distributions, the largest variance is observed when the number of encountering vehicles is one. As the number of encountering vehicles increases, the variance gradually decreases. This is because the estimation result depends on whether the encounter occurs in the early or late stage of the cyclist’s movement when the cyclist encounters only one vehicle. In addition, Figure 23 shows boxplots of the entry-certainty error across all trials. Similar to the trends observed for the range of the confidence interval, the entry-certainty error decreases as the number of encountering vehicles increases. Therefore, these results indicate that increasing the number of encountering vehicles not only reduces estimation uncertainty but also decreases the variability of the framework’s performance.

**Figure 24:**
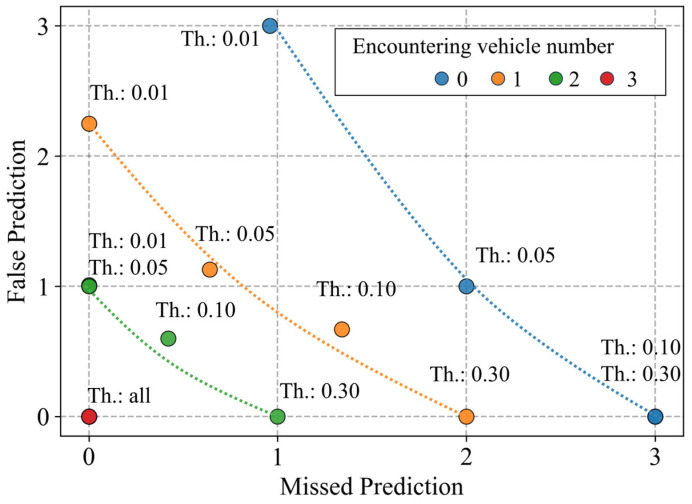
Plot of metrics with respect to intersection-entry prediction.

Finally, Figure 24 summarizes the average numbers of missed entry predictions and false entry predictions. Here, the colors of the markers indicate differences in the number of encountering vehicles. The colored dotted lines represent conceptual trade-off lines. In addition, the thresholds used in each case are indicated by the text label near the marker (e.g., “Th.: 0.01”). Ideally, it is desirable to simultaneously have few missed predictions and few false predictions, and a position in the lower left of this graph indicates more useful predictions. First, we focus on the blue markers, which represent the results for zero encountering vehicles. There is a trade-off between missed and false predictions depending on the thresholds. Therefore, as the threshold increases, missed predictions increase and false predictions decrease. In contrast, as the number of encountering vehicles increases, the position of each marker approaches the lower left. When it reaches three vehicles, both missed and false predictions become zero for all threshold settings. These results indicate that additional observation information improves prediction performance, even with the trade-off between missed and false predictions related to threshold settings.

### 5.3. Case Study on Real Community Road Network

**Figure 25:**
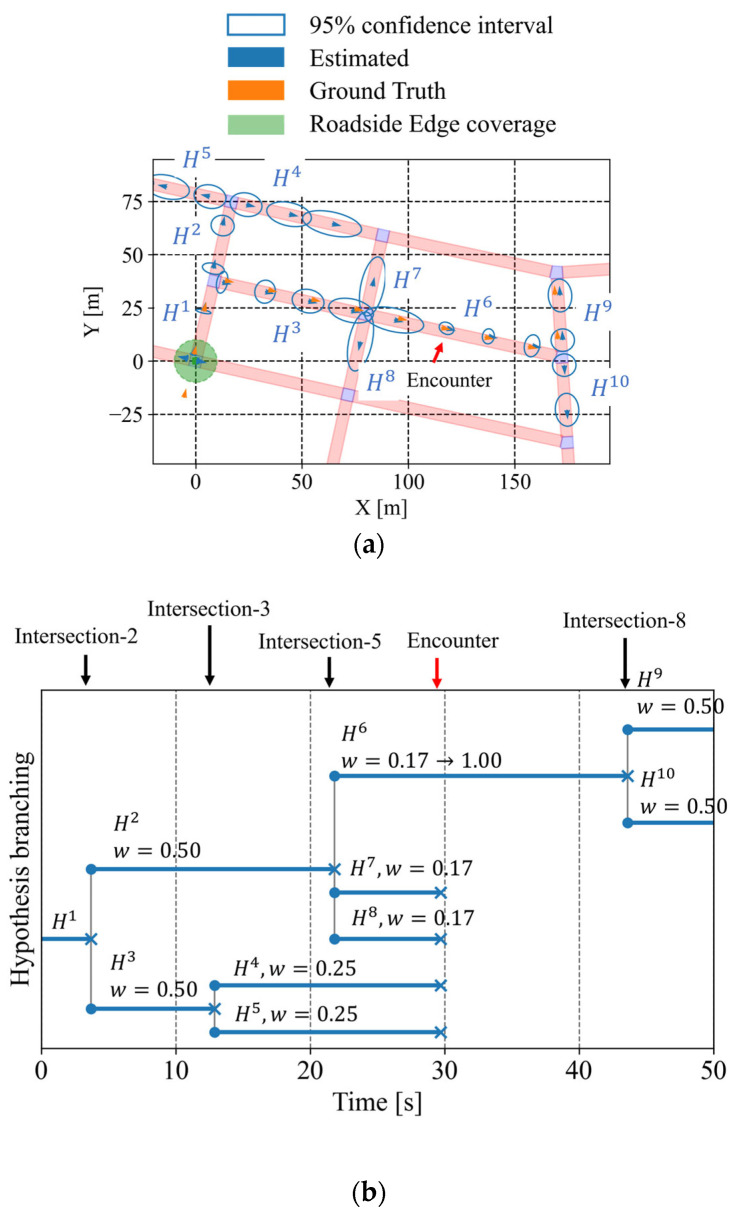
(**a**) Estimated positions according to the framework in the actual community road network. (**b**) Branching of route hypotheses in the actual community road network.

**Figure 26:**
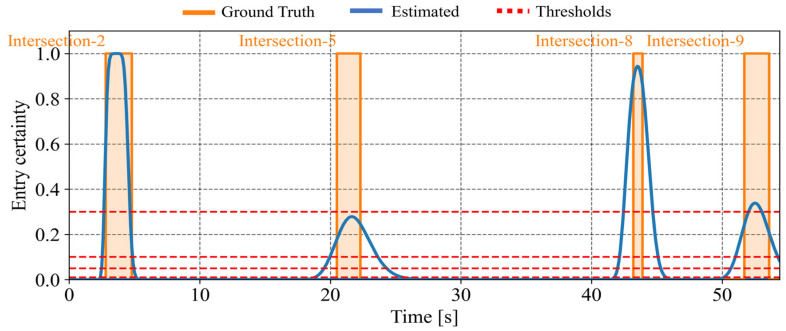
Estimated intersection-entry certainty for each intersection passed by the cyclist in the actual community road network.

Finally, as a case study, we summarize the results obtained by applying the proposed framework to an actual community road network. In this case study, we consider scenarios where the cyclist and one vehicle pass each other. Figure 25a shows the estimated positions according to the framework, and Figure 25b illustrates the branching of route hypotheses in this scenario. In this scenario, the cyclist moves through the bottom-left (Intersection-1), middle-left (Intersection-2), center (Intersection-5), middle-right (Intersection-8), and top-right (Intersection-9) intersections. Although the real community road network has varying road orientations and link lengths unlike the grid network, the result shows that the proposed framework can continue estimation by incorporating road geometry. In addition, Figure 26 illustrates the estimated intersection-entry certainty for each intersection passed by the cyclist. This result indicates that the proposed framework can avoid the complete degradation of the certainty peak and continue intersection-entry prediction throughout the entire road network, despite the limited observation information of a single vehicle encounter. Through this case study, the feasibility of the proposed framework is demonstrated even under real-world road geometry with limited observation information.

## 6. Discussion and Limitations

### 6.1. Usability and User Acceptance of Proposed Framework

In Section 6.1, we discuss the usability of the proposed framework based on the simulation results presented in Section 5. In this study, usability involves two aspects: the quality of an individual intersection-entry prediction and the accuracy of the prediction under multiple route hypotheses. First, we discuss the quality of an individual intersection-entry prediction. Specifically, we consider prediction with a sufficiently low threshold at a single intersection. In this context, “low quality” means that the uncertainty broadens the estimated distribution and the predicted entry time becomes ambiguous. Table 2 presents the confidence interval range of the longitudinal position. As for a no vehicle-encounter scenario, the ranges correspond to approximately 5 s, 14 s, and 26 s for the first, second, and third intersections in TTC, respectively. These ranges, especially 14 s and 26 s, are too wide to be directly used for deterministic intersection-entry prediction. In actual situations, executing deceleration control of vehicles for several seconds may degrade driver acceptance. However, they may remain usable in stochastic collision avoidance strategies based on uncertainty. In the proposed framework, this stochastic approach can be realized with respect to the predicted entry certainty for each intersection. As shown in Figure 19 and Table 2, the entry-certainty error increases as the cyclist proceeds through intersections due to the degraded estimation uncertainty. Nevertheless, even when the entry certainty becomes low, especially at the second and third intersections, the collision avoidance system can adaptively reduce the intervention level based on the certainty and support the driver’s decision-making. For example, the system can send warning messages instead of performing direct control intervention. Therefore, although degradation in the quality of individual intersection-entry predictions is unavoidable under sparse observation conditions, the predictions remain usable through adaptive collision avoidance strategies.

Second, we discuss the accuracy of intersection-entry predictions under multiple route hypotheses. The accuracy of predictions depends on whether the VRU actually follows the route assumed by that hypothesis. From this perspective, we describe the average of the existence ratio within the estimated 95% confidence interval of position, presented in Table 5. As for the network-level average ratio, it exceeds 95% for all encountering vehicle numbers. This result indicates that, on the assumption of a network-level prediction where all active route hypotheses are used without weighting, the proposed framework can estimate the VRU position without critical underestimation. In this situation, missed warnings can be avoided by setting the entry-certainty threshold sufficiently low. In other words, the motion estimation provided by the proposed framework satisfies a necessary condition for preventing missed warnings at the road-network level. As described in Section 1, under environments where available information is sparse, the important point is to avoid missed warnings against hazardous situations involving VRUs at the road-network level. Therefore, the fact that the proposed framework has the potential to prevent missed warnings at the road-network level demonstrates the value of the proposed framework for comprehensive safety functions. On the other hand, such conservative network-level estimation unavoidably possesses concerns regarding false warnings. Here, the branch-level average ratio can be regarded as the proportion of hypotheses that actually correspond to the true VRU’s route among all active hypotheses. From the perspective of individual vehicles who receive warnings, a lower branch-level average ratio implies lower reliability of the warning. In realistic scenarios, the number of vehicles that encounter VRUs during their movement depends on the traffic characteristics of the area. However, it remains unclear how system performance which depends on traffic characteristics affects acceptance of the on-board ADAS from the drivers’ perspective. A similar discussion can be applied to the missed and false entry predictions summarized in Figure 24. Although Section 5.2 showed that additional observation information improves prediction performance, based on this performance, the design of the threshold becomes essential. For example, because this study focuses on avoiding missed predictions, the threshold should be selected from the region on the left side of Figure 24, where missed predictions are low. When the number of encountering vehicles is two or three, setting the threshold to 0.01 or 0.05 results in zero missed predictions. On the other hand, when only zero or one vehicle encounters a VRU, using a low threshold increases false predictions to a non-negligible level. In such cases, as mentioned earlier, it is necessary to reduce the intervention level and support the driver’s decision-making. In summary, system design must consider both user acceptance and traffic characteristics of the target area to ensure practical applicability.

### 6.2. Limitations and Future Prospects

In Section 6.2, we organize the remaining limitations of the proposed framework and discuss prospects for future extension. First, although this study assumed the presence of only a single VRU in the environment to focus on the core behavior of the proposed framework, multiple VRUs may simultaneously exist in realistic scenarios. To address the requirement of data association among multiple VRUs, we previously developed a data association method [47] based on Joint Probabilistic Data Association, which uses sparsely placed roadside sensors. Although this method utilizes the observable area of fixed roadside sensors, it can be extended to vehicles’ on-board sensors. Such an extension would enable the framework to manage multiple VRUs on road networks.

Second, the framework was mainly evaluated on a 3 × 3 road network. The assumption that each road segment has a length of 100 m is considered reasonable, and the assumption of having one observation source within such a 3 × 3 region is not unrealistic. However, the number of route hypotheses can increase rapidly as the road network becomes larger and more complex. In large-scale networks, naive hypothesis management may lead to excessive computational cost and reduced system usefulness. Therefore, the applicability of the proposed framework has been investigated only for small road networks in this study, and strategies for pruning route hypotheses based on hypothesis weight are necessary in the future study.

Third, this study assumes that all roads are straight segments with uniform width. However, real road environments can include complex geometry such as curved segments. On the other hand, such road geometry variations can be addressed to a certain extent by using pseudo-observations. In this study, a linear equation was employed as a pseudo-observation, as shown in Equation (14). However, if the geometry of a road segment can be expressed as a function of position (x,y), it can also be incorporated into the framework as a pseudo-observation. Similarly, for roads with non-uniform widths, the framework can handle the variations by appropriately configuring the parameter $\sigma_{r}^{width}$ in Equation (18). As described in Section 2.4, geometric attributes such as road shape and width are assumed to be included in intermediate-level map information.

Finally, explicit consideration of the VRU’s acceleration and deceleration is necessary. In the assumed situation of this study, sensor information from VRUs is not available. Therefore, a constant-velocity motion model with zero control input is used. However, in real environments, VRUs are likely to accelerate or decelerate near intersections and during turning. This leads to the requirement to incorporate location-dependent motion characteristics into the framework. To address this limitation, our research team previously proposed a method [48] that extracts motion characteristics about acceleration and deceleration behaviors from GNSS logs. By incorporating such location-dependent motion information, the proposed framework may realize more reliable prediction for realistic VRU movement. Related to this issue, there also remains room for improvement in calculation of hypothesis weights. This study treated all routes branching at intersections as equally likely. Motion characteristics extracted from GNSS logs helps assign more reasonable hypothesis weights.

In summary, the above discussion highlights that the proposed framework can provide conservative VRU movement prediction. However, its practical usefulness needs to be further investigated in terms of user acceptance. In addition, this section clarified several limitations and possible prospects for future extension of this study. By addressing these limitations, the proposed framework can be further improved toward practical deployment of V2N-based collision avoidance systems for VRUs on real-world community road networks.

## 7. Conclusions

This study developed a V2N-based framework to address the challenges of sparse observations and frequent route branching on community roads. Simulation results demonstrated that the proposed framework maintains conservative estimation even under sparse observations. This indicates that critical missed warnings can be avoided even with limited sensors. In addition, when additional observation information from encountering vehicles became available, estimation uncertainty was reduced and intersection-entry prediction was improved. Monte Carlo evaluations in a grid network showed changes in usefulness as the number of encountering vehicles increased. Furthermore, a case study on an actual community road network suggested that the framework could appropriately predict intersection-entry under realistic road networks. These results support the feasibility of the proposed framework on actual community roads, where information sources are limited. The proposed framework will contribute to traffic safety on community roads under limited information sources. In addition, as more information sources such as delivery robots and autonomous taxis become available in the future, the usefulness of the proposed framework will be expected to further improve.

However, some challenges remain to be addressed, as discussed in Section 6. In addition, beyond improving prediction performance, further system design should be discussed from the perspective of user acceptance in real safety applications. Furthermore, issues in real communication systems such as latency and packet loss also need to be investigated to ensure practical deployment. Such considerations are important for the practical deployment of the proposed framework in real-world traffic safety systems.

## Figures and Tables

**Table 1:** Average of the existence ratio within a 95% confidence interval of position in the scenario without any encountering vehicles.

Metric	Value
Network-level	100.0 [%]
Branch-level	49.9 [%]

**Table 2:** Evaluation metrics for each intersection in the scenario without any encountering vehicles.

Metric	First Intersection (Intersection-2)	Second Intersection (Intersection-4)	Third Intersection (Intersection-3)
Travel distance from starting intersection	100 [m]	200 [m]	300 [m]
Range of 95% confidence interval of longitudinal position	18.14 [m]	55.87 [m]	105.26 [m]
Entry-certainty error	0.430	0.920	0.985

**Table 3:** Average of existence ratio within a 95% confidence interval of position in the scenario with one encountering vehicle.

Metric	Value
Network-level	100.0 [%]
Branch-level	71.0 [%]

**Table 4:** Evaluation metrics for each intersection in the scenario with one encountering vehicle.

Metric	First Intersection (Intersection-2)	Second Intersection (Intersection-4)	Third Intersection (Intersection-3)
Travel distance from starting intersection	100 [m]	200 [m]	300 [m]
Range of 95% confidence interval of longitudinal position	18.14 [m]	8.86 [m]	40.24 [m]
Entry-certainty error	0.431	0.248	0.899

**Table 5:** Average of the existence ratio within the 95% confidence interval of position.

Metric	Encountering Vehicle Number	Value
Network-level	0	99.97 [%]
	1	99.07 [%]
	2	98.04 [%]
	3	97.89 [%]
Branch-level	0	39.48 [%]
	1	56.19 [%]
	2	69.20 [%]
	3	81.11 [%]

**Figure A1:**
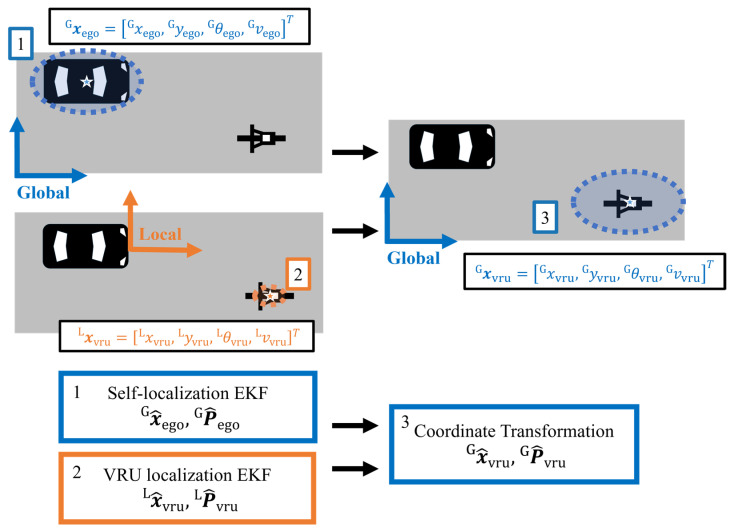
Architecture of Vehicle Edges.

## Data Availability

The original contributions presented in this study are included in the article.

## Abbreviations

The following abbreviations are used in this manuscript.

ADAS	Advanced Driver Assistance System
CI	Covariance Intersection
EKF	Extended Kalman Filter
GNSS	Global Navigation Satellite System
HD maps	High-definition maps
IMU	Inertial Measurement Unit
TTC	Time-To-Collision
UKF	Unscented Kalman Filter
VRU	Vulnerable Road User
V2N	Vehicle to Network

## Appendix A

Here, we describe the details of the formulation of EKFs. Based on the designed state variables in Equations (4) and (5), the transition function f from t−1 to t is defined as follows:

(A1) $$\left[\begin{array}{c} \begin{array}{c} x_{t}\\ y_{t} \end{array}\\ \theta_{t}\\ v_{t} \end{array}\right]=f\left(x_{t-1},u_{t-1}\right)=\left[\begin{array}{c} x_{t-1}+v_{t-1}\Delta t\cos\left(\theta_{t-1}\right)+\frac{1}{2}a_{t-1}\Delta t^{2}\cos\left(\theta_{t-1}\right)\\ {y_{t-1}+v}_{t-1}\Delta t\sin\left(\theta_{t-1}\right)+\frac{1}{2}a_{t-1}\Delta t^{2}\sin\left(\theta_{t-1}\right)\\ \theta_{t-1}+\omega_{t-1}\Delta t\\ v_{t-1}+a_{t-1}\Delta t \end{array}\right]$$

where Δt denotes the time step from t−1 to t. Because f has a nonlinearity caused by the yaw angle, EKF calculates the transition matrix F and the control matrix B by linearizing f as follows:

(A2) $$F_{t-1}=\frac{\partial f\left(x_{t-1},u_{t-1}\right)}{\partial x_{t-1}}=\left[\begin{array}{cccc} 1 & 0 & {-v}_{t-1}\Delta t\sin\left(\theta_{t-1}\right)-\frac{1}{2}a_{t-1}\Delta t^{2}\sin\left(\theta_{t-1}\right) & \Delta t\cos\left(\theta_{t-1}\right)\\ 0 & 1 & v_{t-1}\Delta t\cos\left(\theta_{t-1}\right)+\frac{1}{2}a_{t-1}\Delta t^{2}\cos\left(\theta_{t-1}\right) & \mathrm{\Delta t}\sin\left(\theta_{t-1}\right)\\ 0 & 0 & 1 & 0\\ 0 & 0 & 0 & 1 \end{array}\right]$$

(A3) $$B_{t-1}=\frac{\partial f\left(x_{t-1},u_{t-1}\right)}{\partial u_{t-1}}=\left[\begin{array}{cc} 0 & \frac{1}{2}\Delta t^{2}\cos\left(\theta_{t-1}\right)\\ 0 & \frac{1}{2}\Delta t^{2}\sin\left(\theta_{t-1}\right)\\ \Delta t & 0\\ 0 & \Delta t \end{array}\right]$$

For state estimation, EKF iteratively performs two steps over time: the prediction step and update step. Through this iterative process, EKF calculates estimated state $\hat{x}$ and estimated error covariance matrix $\hat{P}$. First, the prediction step estimates the target’s states using a transition function, as follows:

(A4) $${\hat{x}}_{t|t-1}=f\left({\hat{x}}_{t-1|t-1},u_{t-1}\right)$$

(A5) $${\hat{P}}_{t|t-1}=F_{t-1}{\hat{P}}_{t-1|t-1}F_{t-1}^{T}+B_{t-1}QB_{t-1}^{T}$$

Here, ***Q*** indicates the process noise covariance matrix associated with the control input.

Second, the update step incorporates observation information into the estimation, as follows:

(A6) $$K_{t-1}={\hat{P}}_{t|t-1}{H_{t}}^{T}{\left(H_{t}{\hat{P}}_{t|t-1}{H_{t}}^{T}+R_{t}\right)}^{-1}$$

(A7) $${\hat{x}}_{t|t}={\hat{x}}_{t|t-1}+K_{t-1}\left(z_{t-1}-H_{t}{\hat{x}}_{t|t-1}\right)$$

(A8) $${\hat{P}}_{t|t}=\left(I-K_{t-1}H_{t}\right){\hat{P}}_{t|t-1}{\left(I-K_{t-1}H_{t}\right)}^{T}+K_{t-1}R_{t}K_{t-1}^{T}$$

where ***K*** denotes the Kalman gain, ***H*** the observation matrix, ***z*** the observation, and ***R*** the observation noise matrix. The values of ***H***, ***z***, and ***R*** are determined by observation information provided at each update step. In addition, Equation (A8) is formulated to ensure the symmetry of the matrices and to improve the stability of numerical calculation [49].

Although the general EKF formulation is described above, several parameters need to be specified for the simulations. To determine the parameters, we first define the sensor specifications assumed in this study. Table A1 and Table A2 show the specifications of vehicles’ on-board sensors for self-localization and VRU localization, respectively. These specifications are determined by assuming widespread Advanced Driver Assistance System (ADAS)-level vehicles. In particular, the specification of a millimeter-wave radar for VRU localization is defined with reference to the datasheet of ESR 2.5 [50]. In addition, Table A3 shows the specifications of the roadside sensors. Because we assume that the Roadside Edges are designed to observe traffic participants within intersections, we determine that VRUs’ positions within the intersection are obtained by roadside LiDAR. VRU localization using LiDAR is assumed to rely on object detection based on point cloud data. In this study, the position accuracy of LiDAR-based localization is set to 0.10 m with reference to the analysis results presented in [51], which analyzed VRU localization accuracy using LiDAR point cloud.

**Table A1:** Specifications of vehicles’ on-board sensors for self-localization.

Sensor Type	Observation Frequency	Observation Information	Accuracy (1σ)
GNSS	1 Hz	Position	4.25 m
Speed Sensor	10 Hz	Velocity	0.28 m/s
IMU	10 Hz	Yawing rate	0.01 rad/s
		Acceleration	0.10 m/s^2^

**Table A2:** Specifications of vehicles’ on-board sensors for VRU localization.

Sensor Type	Observation Frequency	Observation Information	Accuracy (1σ)
Millimeter-wave radar	10 Hz	Distance	0.25 m (Noise) ± 5% (Bias)
		Azimuth	0.02 rad
		Velocity	0.12 m/s

**Table A3:** Specification of roadside sensors.

Sensor Type	Observation Frequency	Observation Information	Accuracy (1σ)
LiDAR	10 Hz	Position	0.10 m

Next, we describe two parameters for the prediction step: time step Δt and process noise matrix Q. As for time step Δt, EKFs operate with Δt=0.1 s. As for process noise matrix Q, Table A4 summarizes the elements of Q. Here, two different configurations are defined: one for vehicles and one for cyclists. In the proposed framework, the former is used for the self-localization EKF in the Vehicle Edges, while the latter is used for all other EKFs. As for the vehicles, because the control input can be directly obtained from the on-board IMU, the value of Q is configured based on the sensor accuracy of the IMU. In contrast, control inputs are not observable for the cyclist. Therefore, the prediction step of EKFs for cyclists assumes a zero control input ***u*** = **0**, and the value of Q is defined as the deviation between the assumed constant velocity model and actual behavior. Specifically, the yawing rate ω is treated as equivalent to the steering angular velocity of cyclists, which is approximately 30 deg/s (0.52 rad/s) [52]. As for acceleration *a*, its standard deviation is set to 0.20 m/s^2^, corresponding to one-tenth of the maximum acceleration of 2.0 m/s^2^ [53].

**Table A4:** Elements of Q in EKFs for vehicles and cyclists.

Target	Process Noise Matrix
Vehicle	$Q=\left[\begin{array}{cc} 0.01^{2} & 0\\ 0 & 0.10^{2} \end{array}\right]$
Cyclist	$Q=\left[\begin{array}{cc} 0.52^{2} & 0\\ 0 & 0.20^{2} \end{array}\right]$

Third, we describe values of ***H***, ***z***, and ***R***. Table A5 summarizes the parameters used for vehicle self-localization. In addition, we describe the parameters for VRU localization using millimeter-wave radar. Let the observed relative distance, relative bearing, and relative velocity measured by the millimeter-wave radar be denoted as $d_{rel},\varphi_{rel},v_{rel}$, respectively. Then, the observation is defined as follows:

(A9) $$z=[\begin{array}{c} x\\ y\\ v \end{array}]=[\begin{array}{c} d_{rel}\cos\varphi_{rel}\\ d_{rel}\sin\varphi_{rel}\\ v_{rel} \end{array}]$$

Based on this definition, the Jacobian of the observation model is derived, and the observation noise covariance matrix is computed as follows.

(A10) $$R=[\begin{array}{ccc} \cos\varphi_{rel} & {-d}_{rel}\sin\varphi_{rel} & 0\\ \sin\varphi_{rel} & d_{rel}\cos\varphi_{rel} & 0\\ 0 & 0 & 1 \end{array}][\begin{array}{ccc} {\left(0.25+0.05\times d_{rel}\right)}^{2} & 0 & 0\\ 0 & 0.02^{2} & 0\\ 0 & 0 & 0.12^{2} \end{array}]{\left[\begin{array}{ccc} \cos\varphi_{rel} & {-d}_{rel}\sin\varphi_{rel} & 0\\ \sin\varphi_{rel} & d_{rel}\cos\varphi_{rel} & 0\\ 0 & 0 & 1 \end{array}\right]}^{T}$$

**Table A5:** Values of ***H***, ***z***, and ***R*** used for vehicle self-localization.

Sensor Type	Observation	Observation Matrix	Sensor Noise Matrix
GNSS	$z=\left[\begin{array}{c} x\\ y \end{array}\right]$	$H=\left[\begin{array}{cccc} 1 & 0 & 0 & 0\\ 0 & 1 & 0 & 0 \end{array}\right]$	$R=\left[\begin{array}{cc} 0.10^{2} & 0\\ 0 & 0.10^{2} \end{array}\right]$
Speed Sensor	$z=\left[v\right]$	$H=\left[\begin{array}{cccc} 0 & 0 & 0 & 1 \end{array}\right]$	$R=\left[0.28^{2}\right]$

Finally, we describe pseudo-observation parameters $\sigma_{r}^{width}$ and $\sigma_{r}^{ori}$. Table A6 summarizes the values of pseudo-observation parameters $\sigma_{r}^{width}$ and $\sigma_{r}^{ori}$. The parameters are configured to ensure that the converged 95% confidence interval of the lateral position remains wider than the road width after repeated execution of the prediction step and the pseudo-observation update.

**Table A6:** Values of the pseudo-observation parameters.

Observation Type	Observation Frequency	Observation Uncertainty
Road width	10 Hz	$\sigma_{r}^{width}=15.73\;[m]$
Road orientation	10 Hz	$\sigma_{r}^{ori}=$ 0.79 [deg]

## Appendix B

Here, we briefly describe the outline of the architecture of Vehicle Edges [45]. Figure A1 illustrates the architecture of Vehicle Edges. In each Vehicle Edge, two independent EKFs are executed: one for vehicle self-localization in the global coordinate system and the other for estimating VRU movement in the local coordinate system. The VRU’s estimation is finally obtained by coordinate transformation of the results from these two estimations.

In the following, the superscript on the upper left indicates the coordinate system; “G” denotes the global coordinate system and “L” denotes the local coordinate system. In addition, the superscript on the upper right indicates the attributes; “ego” denotes the vehicle’s state and “vru” denotes the VRU’s state. Therefore, the vehicle’s global state xegoG and the VRU’s local state xvruL are denoted as follows:

(A11) xegoG=xegoG,yegoG,θegoG,vegoGT

(A12) xvruL=xvruL,yvruL,θvruL,vvruLT

On the assumption that the vehicle and the VRU pass each other on a straight road, the coordinate transformation is expressed as follows:

(A13) xvruG=TxegoG,xvruL=xegoG+xvruLcosθegoG−yvruLsinθegoGyegoG+xvruLsinθegoG+yvruLcosθegoGθegoG+θvruLvegoG2+vvruL2+2vegoGvvruLcosθvruLT

From the two EKFs in the Vehicle Edge, estimations (x^egoG, P^egoG) and (x^vruL,P^vruL) are obtained. The mean of the transformed state is calculated via Equation (A13) as x^vruG=Tx^egoG,x^vruL. Then, based on the assumption that x^egoG and x^vruL are statistically independent, covariance matrix P^vruG is approximately calculated by propagating covariance matrices P^egoG and P^vruL through the Jacobians of the coordinate transformation, as follows:

(A14) P^vruG=∂T∂xegoGxegoG=x^egoG,xvruL=x^vruLP^egoG∂T∂xegoGTxegoG=x^egoG,xvruL=x^vruL+∂T∂xvruLTxegoG=x^egoG,xvruL=x^vruLP^vruL∂T∂xvruLTxegoG=x^egoG,xvruL=x^vruL

(A15) ∂T∂xegoG=10−xvruLsinθegoG−yvruLcosθegoG001xvruLcosθegoG−yvruLsinθegoG00010000vegoG+vvruLcosθvruLvvruG

(A16) ∂T∂xvruL=cosθegoG−sinθegoG00sinθegoGcosθegoG00001−vegoGvvruLsinθvruLvvruG000vvruL+vegoGcosθvruLvvruG

where vvruG=vegoG2+vvruL2+2vegoGvvruLcosθvruL.

## Appendix C

Here, we compare the CI fusion used in the proposed framework with a naive fusion approach based on an ordinal EKF. Let $\left({\hat{x}}_{t}^{k},{\hat{P}}_{t}^{k}\right)\;$ denote an external estimation result provided by a Vehicle Edge or a Roadside Edge. The CI fusion used in the proposed framework is formulated in Equations (12) and (13). In contrast, the naive EKF fusion treats each external estimation as an observation in the update step (Equations (A6)–(A8)) by setting H=I, $z={\hat{x}}_{t}^{k}$, and $R={\hat{P}}_{t}^{k}$.

To evaluate the effect of the CI fusion, we conduct Monte Carlo simulations under the same conditions as those described in Section 5.2. Here, we replace the CI fusion with the naive EKF fusion. Table A7 summarizes the average of the network-level existence ratio within the 95% confidence interval of position. The values of the CI fusion are identical to those presented in Table 5. When the number of encountering vehicles is two or three, the existence ratio is below 95%. This result indicates that the naive fusion underestimates the uncertainty compared with the CI fusion. As described in Section 3.4.3, this is because the estimation results provided by each Edge are not statistically independent over time. In other words, if we use naive fusion, the framework repeatedly inputs EKF results into another EKF, which causes double counting of information.

**Table A7:** Average of the existence ratio within the 95% confidence interval of position (network-level).

Fusion Approach	Encountering Vehicle Number	Value
CI fusion	0	99.97 [%]
	1	99.07 [%]
	2	98.04 [%]
	3	97.89 [%]
Naive fusion	0	99.07 [%]
	1	97.02 [%]
	2	93.90 [%]
	3	92.36 [%]

## References

[1] Cabinet Office (2021). The Traffic Safety Basic Plan. https://www8.cao.go.jp/koutu/kihon/keikaku11/pdf/kihon_keikaku_en.pdf.

[2] Geospatial Information Authority of Japan (2019). The Aerial Photograph by Geospatial Information Authority of Japan.

[3] Abdi B., Mirzaei S., Adl M., Hidajat S., Emadi A. (2025). Advancing Vulnerable Road Users Safety: Interdisciplinary Review on V2X Communication and Trajectory Prediction. IEEE Trans. Intell. Transp. Syst..

[4] Dhondge K., Song S., Choi B.-Y., Park H. WiFi-Honk: Smartphone-Based Beacon Stuffed WiFi Car2X-Communication System for Vulnerable Road User Safety. Proceedings of the IEEE 79th Vehicular Technology Conference (VTC Spring).

[5] Hussein A., García F., Armingol J.M., Olaverri-Monreal C. P2V and V2P Communication for Pedestrian Warning on the Basis of Autonomous Vehicles. Proceedings of the IEEE 19th International Conference on Intelligent Transportation Systems (ITSC).

[6] Liu W., Muramatsu S., Okubo Y. Cooperation of V2I/P2I Communication and Roadside Radar Perception for the Safety of Vulnerable Road Users. Proceedings of the 16th International Conference on Intelligent Transportation Systems Telecommunications (ITST).

[7] Barmpounakis S., Tsiatsios G., Papadakis M., Mitsianis E., Koursioumpas N., Alonistioti N. (2020). Collision Avoidance in 5G Using MEC and NFV: The Vulnerable Road User Safety Use Case. Comput. Netw..

[8] Malinverno M., Avino G., Casetti C., Chiasserini C.F., Malandrino F., Scarpina S. (2020). Edge-Based Collision Avoidance for Vehicles and Vulnerable Users: An Architecture Based on MEC. IEEE Veh. Technol. Mag..

[9] Teixeira P., Sargento S., Rito P., Luís M., Castro F. (2023). A Sensing, Communication and Computing Approach for Vulnerable Road Users Safety. IEEE Access.

[10] Wies H., Steinmaßl M., Wagner A., Frühwirth E., Bissinger F., Paier A., Rehrl K., Kölbl R., Oberweger F.F., Zankl C. (2025). Cooperative Collision Risk Detection for C-ITS-Equipped Bicycles and Connected Automated Vehicles. J. Locat. Based Serv..

[11] Wan E.A., Van Der Merwe R. The Unscented Kalman Filter for Nonlinear Estimation. Proceedings of the IEEE Adaptive Systems for Signal Processing, Communications, and Control Symposium.

[12] Arasaratnam I., Haykin S. (2009). Cubature Kalman Filters. IEEE Trans. Autom. Control..

[13] Chen S., Piao L., Zang X., Luo Q., Li J., Yang J., Rong J. (2023). Analyzing differences of highway lane-changing behavior using vehicle trajectory data. Phys. A Stat. Mech. Its Appl..

[14] Kawasaki A., Tasaki T. Trajectory Prediction of Turning Vehicles based on Intersection Geometry and Observed Velocities. Proceedings of the 2018 IEEE Intelligent Vehicles Symposium (IV).

[15] Zhang X., Chen H., Yang W., Jin W., Zhu W. (2021). Pedestrian Path Prediction for Autonomous Driving at Un-Signalized Crosswalk Using W/CDM and MSFM. IEEE Trans. Intell. Transp. Syst..

[16] Li P., Guo H., Bao S., Kusari A. (2023). A Probabilistic Framework for Estimating the Risk of Pedestrian-Vehicle Conflicts at Intersections. IEEE Trans. Intell. Transp. Syst..

[17] Tiger M., Heintz F. Gaussian Process Based Motion Pattern Recognition with Sequential Local Models. Proceedings of the 2018 IEEE Intelligent Vehicles Symposium (IV).

[18] Zhao B., Zhang X., Chen H., Zhu W. (2023). A Novel Prediction Algorithm of Pedestrian Activity Region for Intelligent Vehicle Collision Avoidance System. IEEE Trans. Intell. Veh..

[19] Nayak A., Eskandarian A., Doerzaph Z. (2022). Uncertainty Estimation of Pedestrian Future Trajectory Using Bayesian Approximation. IEEE Open J. Intell. Transp. Syst..

[20] Nayak A., Eskandarian A. (2024). Cooperative Probabilistic Trajectory Prediction Under Occlusion. IEEE Trans. Intell. Veh..

[21] Lambert A., Gruyer D., Saint Pierre G. A fast Montecarlo algorithm for collision probability estimation. Proceedings of the 10th International Conference on Control, Automation, Robotics and Vision.

[22] Houénou A., Bonnifait P., Cherfaoui V. Risk assessment for Collision Avoidance Systems. Proceedings of the 17th International Conference on Intelligent Transportation Systems.

[23] de Campos G.R., Runarsson A.H., Granum F., Falcone P., Alenljung K. Collision avoidance at intersections: A probabilistic threat-assessment and decision-making system for safety interventions. Proceedings of the 17th International Conference on Intelligent Transportation Systems.

[24] Tao L., Watanabe Y., Li Y., Yamada S., Takada H. (2021). Collision Risk Assessment Service for Connected Vehicles: Leveraging Vehicular State and Motion Uncertainties. IEEE Internet Things J..

[25] Gao Z., Bao M., Cui T., Shi F., Chen X., Wen W., Gao F., Zhao R. (2024). Collision Risk Assessment for Intelligent Vehicles Considering Multi-Dimensional Uncertainties. IEEE Access.

[26] Julier S.J., Uhlmann J.K. A Non-Divergent Estimation Algorithm in the Presence of Unknown Correlations. Proceedings of the American Control Conference.

[27] Li H., Nashashibi F., Yang M. (2013). Split Covariance Intersection Filter: Theory and Its Application to Vehicle Localization. IEEE Trans. Intell. Transp. Syst..

[28] Li H., Nashashibi F. (2013). Cooperative Multi-Vehicle Localization Using Split Covariance Intersection Filter. IEEE Intell. Transp. Syst. Mag..

[29] Héry E., Xu P., Bonnifait P. Distributed Asynchronous Cooperative Localization with Inaccurate GNSS Positions. Proceedings of the IEEE Intelligent Transportation Systems Conference (ITSC).

[30] Héry E., Xu P., Bonnifait P. A Study of Different Observation Models for Cooperative Localization in Platoons. Proceedings of the IEEE 26th International Conference on Intelligent Transportation Systems (ITSC).

[31] Cai K., Qu T., Liu F., Chen H., Xie L. (2024). Cooperative Perception With Localization Uncertainty: A Cubature Split Covariance Intersection Framework. IEEE Trans. Intell. Transp. Syst..

[32] Elghazaly G., Frank R., Harvey S., Safko S. (2023). High-Definition Maps: Comprehensive Survey, Challenges, and Future Perspectives. IEEE Open J. Intell. Transp. Syst..

[33] Japan Digital Road Map Association Digital Road Map. https://www.drm.jp/assets/pdf/DRM_Brochure.pdf.

[34] Chiang Y.-Y., Leyk S., Knoblock C.A. (2014). A Survey of Digital Map Processing Techniques. ACM Comput. Surv..

[35] Biagioni J., Eriksson J. (2012). Inferring Road Maps from Global Positioning System Traces: Survey and Comparative Evaluation. Transp. Res. Rec. J. Transp. Res. Board.

[36] Joshi A., James M.R. (2015). Generation of Accurate Lane-Level Maps from Coarse Prior Maps and Lidar. IEEE Intell. Transp. Syst. Mag..

[37] Werling M., Ziegler J., Kammel S., Thrun S. Optimal trajectory generation for dynamic street scenarios in a Frenét Frame. Proceedings of the IEEE International Conference on Robotics and Automation.

[38] Simon D., Chia T.L. (2002). Kalman filtering with state equality constraints. IEEE Trans. Aerosp. Electron. Syst..

[39] Song D., Tharmarasa R., Kirubarajan T., Fernando X.N. (2018). Multi-Vehicle Tracking With Road Maps and Car-Following Models. IEEE Trans. Intell. Transp. Syst..

[40] Kirubarajan T., Bar-Shalom Y., Pattipati K.R., Kadar I. (2000). Ground target tracking with variable structure IMM estimator. IEEE Trans. Aerosp. Electron. Syst..

[41] Krishanth K., Tharmarasa R., Kirubarajan T., Valin P., Meger E. (2014). Prediction and retrodiction algorithms for path-constrained targets. IEEE Trans. Aerosp. Electron. Syst..

[42] Yang C., Blasch E. Track Fusion with Road Constraints. Proceedings of the 10th International Conference on Information Fusion.

[43] Duan Z., Li X.R. (2013). The Role of Pseudo Measurements in Equality-Constrained State Estimation. IEEE Trans. Aerosp. Electron. Syst..

[44] Zhang M., Knedlik S., Loffeld O. An adaptive road-constrained IMM estimator for ground target tracking in GSM networks. Proceedings of the 11th International Conference on Information Fusion.

[45] Watanabe K., Ito T. Initial study on global position and uncertainty estimation for traffic participant observed from moving local coordinate system. Proceedings of the 8th International Symposium on Future Active Safety Technology towards Zero-Traffic Accidents.

[46] SciPy Optimize: Minimize_Scalar. https://docs.scipy.org/doc/scipy/reference/generated/scipy.optimize.minimize_scalar.html.

[47] Ishibashi E., Watanabe K., Ito T. (2025). Sensor Coverage Aware Probabilistic Data Association to Track Multiple Traffic Participants Using Sparsely Placed Roadside Sensors. Int. J. Automot. Eng..

[48] Suzuki K., Ito T. (2025). Virtual Observation Using Location-Dependent Statistical Information of Cyclists’ Movement for Estimation of Position and Uncertainty. Sensors.

[49] Brown R.G., Hwang P.Y.C. (2012). Introduction to Random Signals and Applied Kalman Filtering with Matlab Exercises.

[B50-sensors-26-01229] 50.Delphi Technologies. ESR Quick Specification Sheet. Unpublished technical documentation.

[51] Van Der Sluis J.R., Pool E.A.I., Gavrila D.M. An Experimental Study on 3D Person Localization in Traffic Scenes. Proceedings of the 2020 IEEE Intelligent Vehicles Symposium (IV).

[52] Fonda B., Sarabon N., Li F.X. (2015). Bicycle rider control skills: Expertise and assessment. J. Sports Sci..

[53] Kametani Y., Yamanaka H., Kakihara K., Yokota S. An analysis on characteristics of cycling behavior by elderly people at slopes and in starting to move. Proceedings of the 39th Infrastructure Planning Conference.

